# Evaluation of vaginal microbiome equilibrium states identifies microbial parameters linked to resilience after menses and antibiotic therapy

**DOI:** 10.1371/journal.pcbi.1011295

**Published:** 2023-08-11

**Authors:** Christina Y. Lee, Jenna Diegel, Michael T. France, Jacques Ravel, Kelly B. Arnold

**Affiliations:** 1 Department of Biomedical Engineering, University of Michigan, Ann Arbor, Michigan, United States of America; 2 Institute for Genome Sciences and Department of Microbiology and Immunology, University of Maryland School of Medicine, Baltimore, Maryland, United States of America; Utrecht University, NETHERLANDS

## Abstract

The vaginal microbiome (VMB) is a complex microbial community that is closely tied to reproductive health. Optimal VMB communities have compositions that are commonly defined by the dominance of certain *Lactobacillus* spp. and can remain stable over time or transition to non-optimal states dominated by anaerobic bacteria and associated with bacterial vaginosis (BV). The ability to remain stable or undergo transitions suggests a system with either single (mono-stable) or multiple (multi-stable) equilibrium states, though factors that contribute to stability have been difficult to determine due to heterogeneity in microbial growth characteristics and inter-species interactions. Here, we use a computational model to determine whether differences in microbial growth and interaction parameters could alter equilibrium state accessibility and account for variability in community composition after menses and antibiotic therapies. Using a global uncertainty and sensitivity analysis that captures parameter sets sampled from a physiologically relevant range, model simulations predicted that 79.7% of microbial communities were mono-stable (gravitate to one composition type) and 20.3% were predicted to be multi-stable (can gravitate to more than one composition type, given external perturbations), which was not significantly different from observations in two clinical cohorts (HMP cohort, 75.2% and 24.8%; Gajer cohort, 78.1% and 21.9%, respectively). The model identified key microbial parameters that governed equilibrium state accessibility, such as the importance of non-optimal anaerobic bacteria interactions with *Lactobacillus* spp., which is largely understudied. Model predictions for composition changes after menses and antibiotics were not significantly different from those observed in clinical cohorts. Lastly, simulations were performed to illustrate how this quantitative framework can be used to gain insight into the development of new combinatorial therapies involving altered prebiotic and antibiotic dosing strategies. Altogether, dynamical models could guide development of more precise therapeutic strategies to manage BV.

## Author summary

The vaginal microbiome (VMB) is critical to female reproductive health, and communities characterized by the abundance of certain microbial species are considered more protective against adverse reproductive outcomes. The most optimal communities are dominated by *Lactobacillus* spp., which can remain stable or undergo shifts to an overgrowth of non-optimal vaginal bacteria associated with a common condition called bacterial vaginosis (BV). Despite BV affecting nearly 30% of reproductive age women, the key drivers of community shifts associated with BV and suboptimal treatment outcomes remain poorly understood. Inspired by empirical clinical observations of common community state types, we use quantitative models to identify microbial parameters that underpin variability in community stability across individuals, and gained insight into key inter-species interactions that could be leveraged in new strategies to manage or prevent BV.

## Introduction

The vaginal microbiome is a complex system that plays a fundamental role in women’s health, influencing fertility [1,2], susceptibility to infectious disease [3,4], and drug efficacy [5–7]. An optimal vaginal microbiome is characterized by low microbial diversity and an abundance of *Lactobacillus* species (spp.), which can shift to a non-optimal state associated with a diverse array of anaerobic bacterial spp., commonly *Gardnerella vaginalis*, *Atopobium vaginae* and *Prevotella* spp. [8]. Previously published clinical observations suggest that the VMB gravitates to five main compositions defined by microbial species abundance known as community state types (CSTs): three that are dominated by *Lactobacillus* (LB) species and associated with optimal health (*L*. *crispatus*, CST -I; *L*. *gasseri*, CST -II; *L*. *jensenii*, CST -V); one dominated by *L*. *iners* and associated with an increased transition rate to non-optimal states (CST -III); and a high bacterial diversity state, lacking *Lactobacillus* spp., commonly associated with BV (CST -IV) [8]. Understanding these equilibrium states and associated stability is challenging and limited by a lack of longitudinal studies in humans with frequent (daily) sampling; however, the limited longitudinal studies support a biological system that has set equilibrium compositions where external perturbations impacting the cervicovaginal environment such as menses, sexual or hygienic behaviors and antibiotics, can either transiently impact community composition (relative abundance) or result in dramatic, sustained shifts in composition. These behaviors are hallmarks of communities that gravitate to one equilibrium composition state (mono-stable systems) versus communities that can exhibit more than one equilibrium composition state (multi-stable systems).

The ability for a community to have multiple equilibrium states has been studied in macro-ecology to determine factors driving dramatic composition (regime) shifts after short term environmental changes with hopes these factors could be used to regulate ecosystem composition and function [9,10]. Whether the vaginal microbial community has a single equilibrium state (mono-stable) or multiple equilibrium states (multi-stable) could have important implications in understanding community responses to menses or antibiotic therapies. For example, several studies report changes in the VMB composition during menses, typically characterized by temporary high diversity states lacking *Lactobacillus* spp. However, not all women are affected to the same degree, with some women exhibiting little to no fluctuations which would be indicative of a mono-stable system and others undergoing dramatic, sustained switches in composition indicative of multiple possible equilibrium states [11–13]. Similarly, understanding non-optimal mono-stable systems could help explain the high rate of BV recurrence after standard of care antibiotic, metronidazole or clindamycin, as mono-stable systems would be resilient to the temporary regimen of antibiotics and would require other specific and lasting alterations to prevent recurrent BV episodes [14].

Factors that dictate whether the VMB exists in a mono-stable or multi-stable equilibrium state are likely a mix of host and microbial factors that impact growth characteristics and interactions between species. Both host characteristics and microbial characteristics are highly variable, with individuals of comparable VMB compositions exhibiting variable phenotypes due to complex inter-species interactions working in concert or antagonistically to regulate community composition and function [12]. For example, a common assumption is that certain *Lactobacillus* spp. (including *L*. *crispatus*, *L*. *jensenii* and *L*. *gasseri)* inhibit the growth of BV-associated bacteria by producing compounds like D-lactic acid, L-lactic acid (except *L*. *jensenii*), and bacteriocins [15–17]. Despite this assumption, reports suggest that these species have variable inhibitory strengths and even strains of the same *Lactobacillus* spp. can have vastly differing capabilities at decreasing the abundance of BV-associated bacteria [18]. One *Lactobacillus* sp., *L*. *iners*, does not produce D-lactic acid and is more commonly associated with BV [19]. *L*. *iners* can produce a cytolysin similar to *G*. *vaginalis*, suggesting this *L*. *iners* may play a different role in vaginal ecology than *L*. *crispatus*, *L*. *jensenii*, and *L*. *gasseri*. Additionally, of the core *Lactobacillus* spp., *L*. *iners* is most associated with vaginal dysbiosis [12,20]. Communities associated with BV have high species diversity as well as have the ability to engage in cooperative behavior via cross-feeding or biofilm formation that could influence system stability and susceptibility to antibiotics [21,22]. Like the *Lactobacillus* spp., there is also a high degree of intra-species variability, especially for one of the most commonly observed bacteria with BV, *G*. *vaginalis*, where some species and strains are more associated with recurrent BV and suboptimal treatment outcomes [18,23–25]. The combination of intra-species variability with inter-species ecological interactions complicates assembly of microbial communities and convolutes understanding of BV pathogenesis as well as responsiveness to antibiotic treatment. Considering all these contributing factors, the ability to quantitatively assess how combinations of these variables contribute to community stability (consistency in composition over time) or multi-stability could be essential in understanding VMB composition shifts after menses or antibiotic therapy.

The application of quantitative mathematical models to unravel complexities of inter-species interactions and host-microbiota interactions have demonstrated promise for understanding complex microbial dynamics. Generalized Lotka-Volterra models (gLVM), which represent inter-species interactions with a coefficient that describes pairwise, additive, abundance (density) dependent interaction strengths, have been used to model the gut microbiome [26,27] and in theoretical microbial ecology [28,29]. However, obtaining parameters for these models requires dense longitudinal sampling, absolute abundance data, and population level consistency of species present, which are features that are lacking for *in vivo* studies of the VMB due to inadequate animal models [30], difficulty in clinical sample collection, and unique characteristics of VMB composition, where communities can be nearly completely dominated by a single species [8,11,12,31]. Even when all these conditions are met, the fitting of these models to noisy temporal data can lead to variable results dependent on data pre-processing steps or from assumptions that arise across different model fitting algorithms [32]. As a result, these methods have not yet been used to reveal mechanistic insight into the VMB, which would be especially valuable for understanding which microbial parameters govern mathematical equilibrium states related to VMB CSTs.

Here we use a simplified gLVM of VMB community state types (CSTs) consistently observed across women to understand how variability in microbial parameters may govern equilibrium state accessibility and differences in composition changes after antibiotic therapy or menses. We address challenges related to parameter availability by capturing physiologically relevant variability using a global sensitivity and uncertainty analysis [33] and compare results to clinical equilibrium behavior subtypes determined from two longitudinal clinical studies. By matching clinically observed equilibrium behavior subtypes to parameter spaces that share that same behavior, we can interrogate which parameters differentiate these subtypes and make predictions on how each subtype will respond to external perturbations. This methodology also overcomes challenges associated with inconsistent parameter estimations that arise from fitting gLVMs directly to *in vivo* data. Our goal was to pinpoint key microbial interaction and growth terms that determine mono- vs. multi-stability and drive compositional changes after menses or antibiotic therapy. Identification of such parameters could be used to focus future research regarding specific interspecies interactions that can re-orient a system to an ideal equilibrium behavior subtype, by targeting certain substrates that promote or inhibit the growth of select species, production of lactic acid [34–36], or selective bacteriocins [37]. We also demonstrate how this framework could be used to identify new dosing regimens and combinatorial therapies to improve BV treatment outcomes across heterogenous populations and given individual’s equilibrium behavior subtype.

## Results

### A computational model reveals the potential for the VMB to exist in mono-stable or multi-stable states depending on microbial parameters that vary across individuals

**Fig 1:**
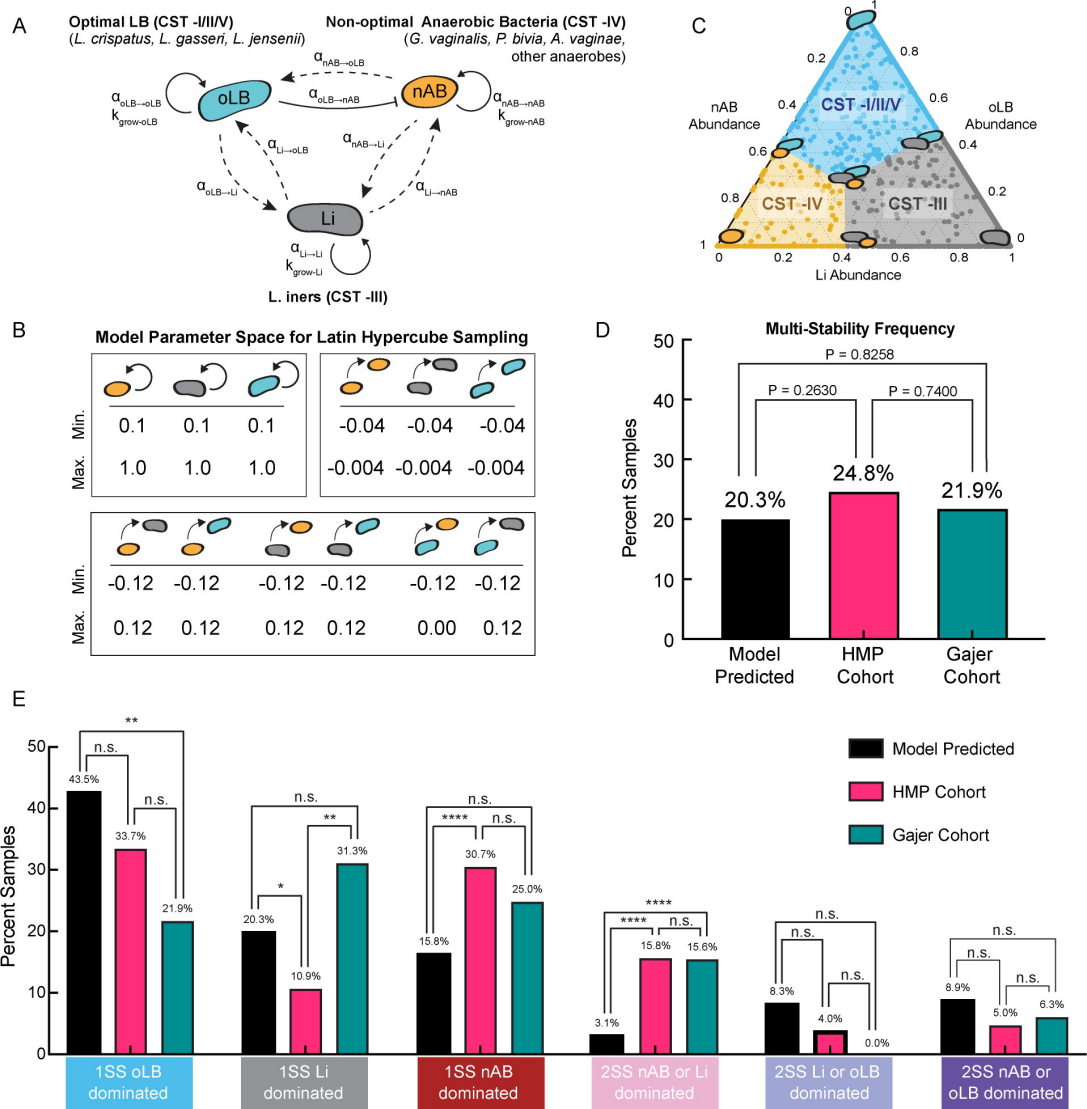
Mathematical model of vaginal community state types. (A) Model schematic of a generalized Lotka-Volterra Model for three community groups: optimal *Lactobacillus* spp. (oLB), *L*. *iners* (Li), and non-optimal anaerobic bacteria (nAB). Model equations capture growth rates, carrying capacity and interaction terms. (B) Parameter ranges used to define uniform distributions for a global uncertainty and sensitivity analysis using Latin Hypercube Sampling (LHS). (C) Mapping of the model predicted steady-state compositions to model defined CSTs using a nearest centroid model based on VALENCIA centroids. (D) Analysis of the overall frequency of multi-stable states from the model predicted versus clinical observations in two cohorts (HMP and Gajer cohorts). Statistical comparisons were made using χ2-tests. (E) Equilibrium frequencies from the base model parameter space predicted and clinical frequencies of the HMP Cohort (N = 101, pink) and the Gajer Cohort (N = 32, teal). Statistical comparisons were made using χ2-tests. P-values were represented as n.s. (not significant, P > 0.05), * (P < 0.05), ** (P < 0.01), *** (P < 0.001), **** (P < 0.0001) and were not adjusted for multiple comparisons.

In order to understand the relationship between microbial parameters and community composition and stability, we created a generalized Lotka-Volterra ODE Model (**gLV**) to predict simplified VMB CSTs over time as a function of microbial growth and interaction characteristics. The simplified CSTs included the “optimal” *Lactobacillus* spp.- dominated state (oLB dominated, combined CST -I, -II, -V), the *Lactobacillus* sp., *L*. *iners-* dominated state (Li dominated, CST -III), and a state with high bacterial diversity, associated with non-optimal anaerobic bacteria (nAB) and BV (nAB dominated, CST -IV; Fig 1A). This model had seven nonzero steady states with the potential for multiple of the seven states to be accessible for a given set of microbial parameters (multi-stability, S1 Text). These seven analytical steady states were related to CSTs through a nearest centroid classifier based on their predicted composition of nAB, Li, and oLB (Fig 1B). The CST classified model steady states, which represent equilibrium behaviors observed clinically, are referred to as equilibrium behavior subtypes depending on the dominating microbe in the community. Equilibrium behavior subtypes describe the composition a microbial community inherently gravitates toward without external perturbations on the community such as menses, antimicrobial therapy, or other host factors. To understand the relationship between microbial growth characteristics and interspecies interactions on the internal stability of VMB community composition, heterogeneity within a physiologically relevant parameter space was generated using Latin Hypercube Sampling (LHS) of uniform distributions defined by experimental and empirical observations for each microbial parameter (S1 Table and Figs 1C and S1). Then, local stability analysis was performed to analytically determine the accessible steady states for each parameter set (simulated sample) generated from the LHS (N = 5,000; S1 Text). Model predictions for community steady states were converted to CSTs (oLB dominated CST -I/II/V, nAB dominated CST -IV, and Li dominated CST -III) using a nearest centroid classifier on the analytically predicted equilibrium composition similar to previously used methodologies to classify CSTs clinically (S2 Table, [31]). This classification method linked predicted *in silico* equilibrium behavior subtypes to clinically observed subtypes at the CST level (S2 Fig). Overall model results demonstrated six, physiologically relevant equilibrium behavior subtypes that were either mono-stable (1SS, one stable state) or multi-stable (>2SS, two or more stable states; Fig 1D and 1E). The most common equilibrium behavior subtypes corresponded to three mono-stable systems: (1) the optimal *Lactobacillus* spp. dominant mono-stable equilibrium subtype (1SS oLB dominated CST -I/II/V; 43.5% of simulated samples), (2) the *L*. *iners* species dominant equilibrium subtype (1SS Li dominated CST -III; 20.3% of simulated samples), and (3) the nAB species dominated (BV-associated) equilibrium subtype (1SS nAB dominated CST -IV; 15.8% of simulated samples; Fig 1E). To compare model predictions to clinical equilibrium behavior frequencies, clinical equilibrium behavior subtypes were determined from two longitudinal cohorts, the Human Microbiome Project Cohort (HMP Cohort, N = 101 patients) [12] and the Gajer et al. 2012 cohort (Gajer cohort, N = 32 patients [11]; Fig 1E). Equilibrium behavior subtypes were calculated from each patients’ time series by calculating a CST transition matrix (S2 Fig). For example, patients whose transition matrix was primarily associated with within-state transitions (i.e. CST -I/II/V to CST -I/II/V) were classified as mono-stable (1SS oLB dominated; S2 Fig), whereas patients whose transition matrix had two cases of high within state transitions rates (for example, 65% were CST -IV to CST -IV and 31% were CST -I/II/V to CST -I/II/V) were classified as multi-stable (2SS nAB dominated/oLB dominated; S2 Fig). There was no significant difference in the frequency of predicted multi-stable vaginal communities compared to the clinical data (predicted 20.3% vs. 24.8% in the HMP cohort and 21.9% in the Gajer cohort; P = 0.2630 and 0.8258, respectively; Fig 1D). For specific equilibrium subtypes, frequencies differed both between the two clinical cohorts and the model predictions (Fig 1E). The model tended to underestimate nAB dominated equilibrium subtypes in favor of oLB dominated equilibrium subtypes, with the 2SS nAB dominated/Li dominated subtype having the most consistent discrepancy when compared to the two clinical cohorts (Fig 1E). The underestimation of subtypes that exhibit nAB dominated compositions is likely due to constraining the interaction of oLB on nAB to be negative (inhibitory) in the LHS parameter distributions, favoring oLB dominated equilibrium subtypes. Calibration of the parameter distributions to be more representative of a given population could increase the predictive power of the model when external perturbations are simulated. Overall, this analysis supports that the vaginal microbiome can exist as either a mono-stable or a multi-stable system, and the predominant state can be replicated by the specific growth and microbial interaction parameters that vary across women.

### Mono-stability is driven by specific interspecies interaction terms

**Fig 2:**
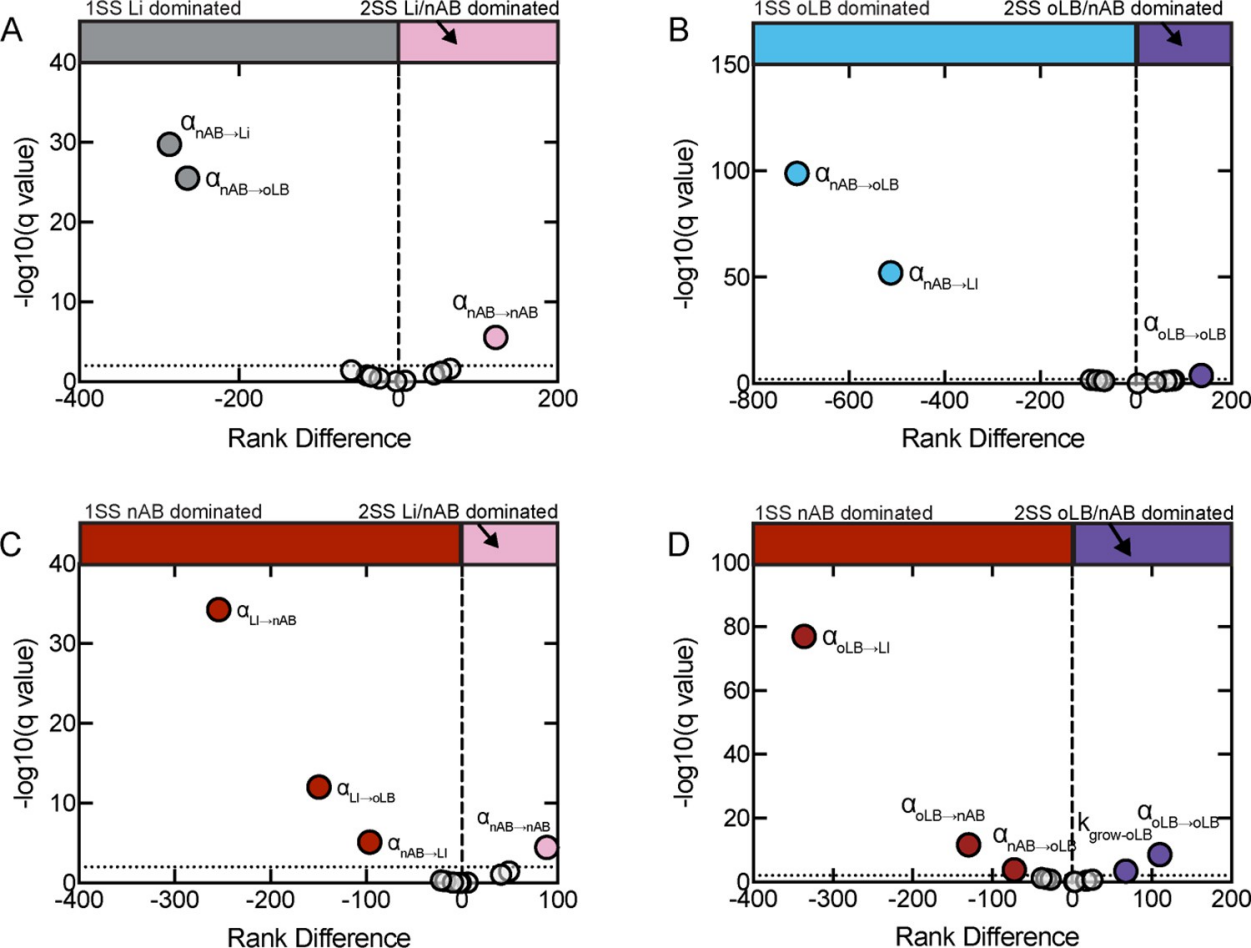
Assessment of parameters that drive multi-stable vs mono-stable states. Volcano plots from multiple Wilcoxon rank sum tests with FDR-adjusted p-values. Colored points indicate parameters that were significantly different between comparison groups. (A) 1SS Li dominated (gray) vs 2SS Li dominated/nAB dominated (pink). (B) 1SS oLB dominated (blue) vs 2SS oLB dominated/nAB dominated (purple) (C) 1SS nAB dominated (red) vs 2SS nAB dominated/Li dominated (pink) (D) 1SS nAB dominated (red) vs 2SS nAB dominated/oLB dominated (purple).

To understand microbial parameters that drive mono-stable vs. multi-stable communities, a comparison of the parameters sets associated with different equilibrium behavior subtypes was completed using multiple Wilcoxon rank sum tests with FDR-adjusted p-values (Fig 2). This analysis characterized the inherent stability of the microbial community to gravitate toward one equilibrium composition or be able to switch between two equilibrium compositions, which helps define whether the community will undergo compositional shifts given an external perturbation such as sexual and hygienic behaviors, menses, or antibiotic therapies (S3 Table). The comparison of mono-stable optimal subtypes (1SS Li dominated and 1SS oLB dominated) to multi-stable optimal/non-optimal subtypes (2SS Li dominated/nAB dominated and 2SS oLB dominated/nAB dominated revealed that the interaction of nAB with Li and oLB (α_nAB→Li_, α_nAB→oLB_) was significantly associated with mono-stable subtypes (Fig 2A and 2B). This result indicates the effect nAB have on *Lactobacillus* spp. could dictate inherent community stability from external perturbations. This observation is notable, as there are few studies that describe the impact nAB on *Lactobacillus* spp. (oLB or Li). The comparison of the stable non-optimal subtypes (1SS nAB dominated) to multi-stable subtypes (2SS nAB dominated/Li dominated and 2SS nAB dominated/oLB dominated) supported the importance of the associated *Lactobacillus* spp. in driving mono-stability, where weaker interactions between oLB or Li with nAB were associated with the 1SS nAB dominated group (α_Li→nAB_,α_oLB→nAB_; Fig 2C and 2D). Additionally, the interactions between *Lactobacillus* spp. were associated with the 1SS nAB dominated group, with more positive interactions between *Lactobacillus* spp. associated with the 1SS nAB dominated communities (α_Li→oLB_,α_oLB→Li_; Fig 2C and 2D). Pairwise *Lactobacillus* spp. interactions are thus a potential non-intuitive target for altering microbial community equilibrium behavior, as interactions between species like *L*. *iners* and *L*. *crispatus*, *L*. *jensenii* or *L*. *gasseri* remain poorly characterized. Altogether these results indicate that drivers of mono- vs multi-stability are specific to equilibrium subtypes and indicate that inter-species interactions are drivers of mono-stable versus multi-stable states.

### Equilibrium subtypes may explain variability in temporary vs. sustained transitions observed after menses

The vaginal microbiota is subject to external and internal factors that dictate whether the community will undergo changes in composition over time. One common perturbation is menses, where during menses studies report changes in VMB composition, typically a temporary change from low diversity, *Lactobacillus* spp. dominance, to high diversity states associated with BV. Notably, reports indicate variability in the degree of impact of menses on composition, ranging from no noticeable impact on VMB composition to individuals undergoing dramatic and sustained shifts after menses [11–13]. To explore the impact of menses on initially optimal microbial communities, equilibrium behavior subtypes that could exhibit oLB or Li dominance were evaluated (1SS oLB dominated, 1SS Li dominated, 2SS oLB dominated/nAB dominated, 2SS Li dominated/oLB dominated). To be able to assess model predictions to clinical data at a population level, an *in silico* HMP cohort was created by resampling the base parameter space and matching the equilibrium behavior distribution exhibited by the HMP cohort (S3 Fig). The parameters were also scaled to be on the time scale observed for growth rates and interaction terms observed in a murine gut microbiome model [26]. Menses was simulated based on the connection between elevated levels of certain biogenic amines during menstruation and their associated connection with transitions to BV positive states [38]. These biogenic amines were reported to alter *Lactobacillus* spp. characteristics *in vitro* including decreased growth rates of *Lactobacillus* spp. (k_grow-Li_, k_grow-oLB_) and decreased production of lactic acid (less inhibitory α_Li→nAB_ and α_oLB→nAB_).

**Fig 3:**
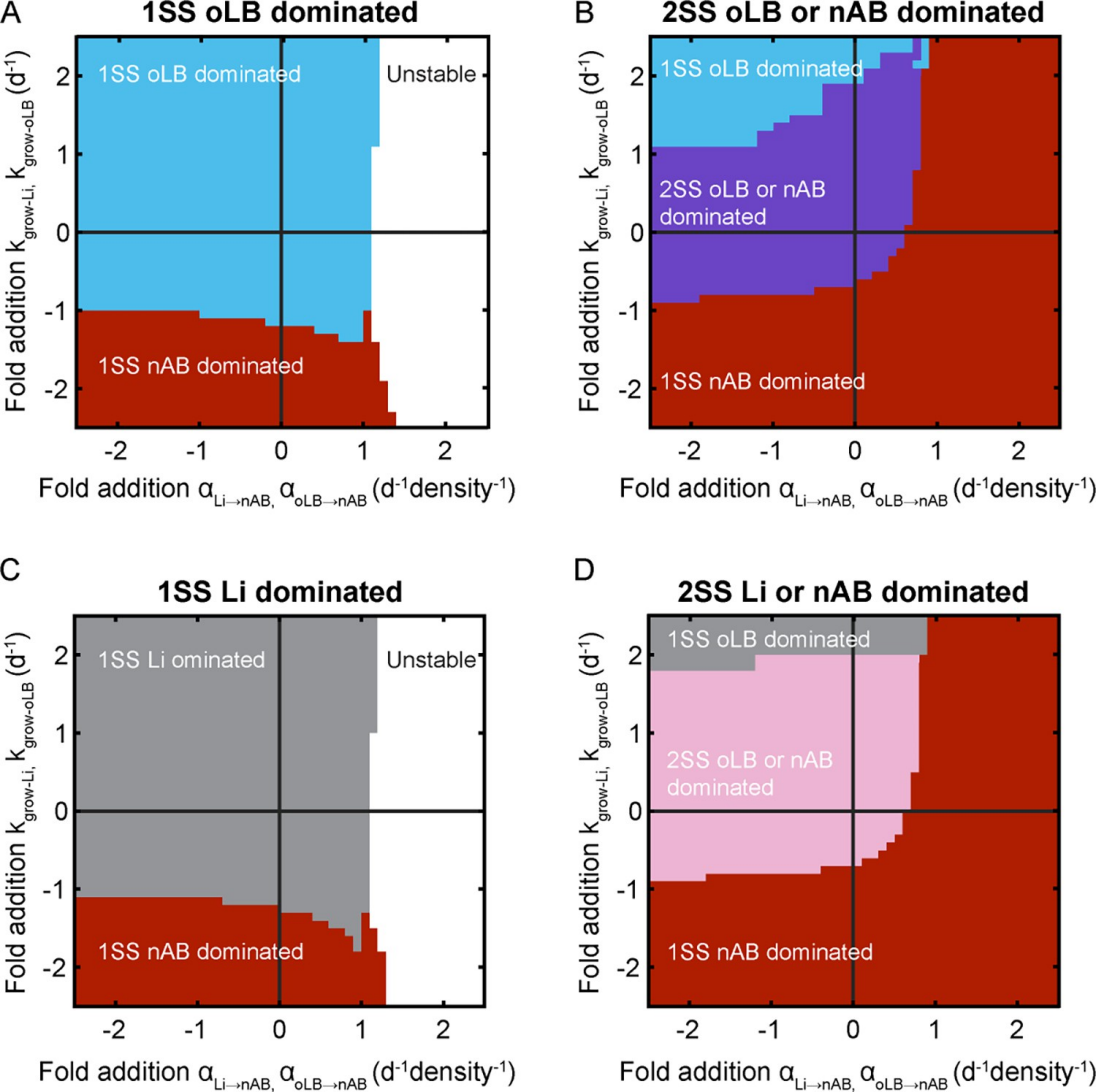
Bifurcation analysis to explore menses-associated parameter alterations. Bifurcations were completed on each simulated sample for a given equilibrium behavior. Bifurcation plots represent the predicted equilibrium behavior subtype over a range of parameter changes. The most frequently observed equilibrium behavior across all simulated samples for a given parameter combination is plotted. Parameter changes are represented as a fold addition from the original parameter value. For example, the origin (0,0) indicates the baseline values for each sample and (-1,0.5) would indicate a 100% decrease in α_oLB →nAB and_ α_Li →nAB_ and a 50% increase in k_grow-Li_ and k_grow-oLB_. Menses-associated changes are in the lower right quadrant (decrease in the growth of oLB and Li, less negative interactions between oLB and Li on nAB. (A) 1SS oLB dominated (N = 388) (B) 2SS oLB dominated/nAB dominated (N = 58) (C) 1SS Li dominated (N = 123) (D) 2SS Li dominated/nAB dominated (N = 186) equilibrium subtypes.

First, a two-dimensional sensitivity (bifurcation) analysis was used to assess how menses-related parameter changes could shift steady-state accessibility across the four *Lactobacillus* spp. dominated equilibrium behavior subtypes. The global bifurcation analysis of the 1SS oLB dominated and the 2SS oLB dominated/nAB dominated equilibrium behavior subtypes demonstrated that the mono-stable groups were resilient to changes in menses-affected parameters, requiring growth rates of oLB to be inhibited to point where these populations would be actively dying (negative; Fig 3A). In contrast, the multi-stable simulated samples required less significant inhibition of oLB/Li growth and could be switched to a 1SS nAB dominated equilibrium behavior with only increases in the interaction of oLB and Li. on nAB (α_Li→nAB_ and α_oLB→nAB_; Fig 3B). These trends were mirrored in the global bifurcation of the 1SS Li dominated and the 2SS Li dominated/nAB dominated subtypes where the mono-stable groups were more resilient to changes in equilibrium behavior, and the multi-stable systems were more sensitive to switching to the 1SS nAB dominated equilibrium behavior (Fig 3C and 3D). These results demonstrate that multi-stable communities are more likely to switch to nAB dominated equilibrium subtypes for the same alteration of parameters than mono-stable communities.

**Fig 4:**
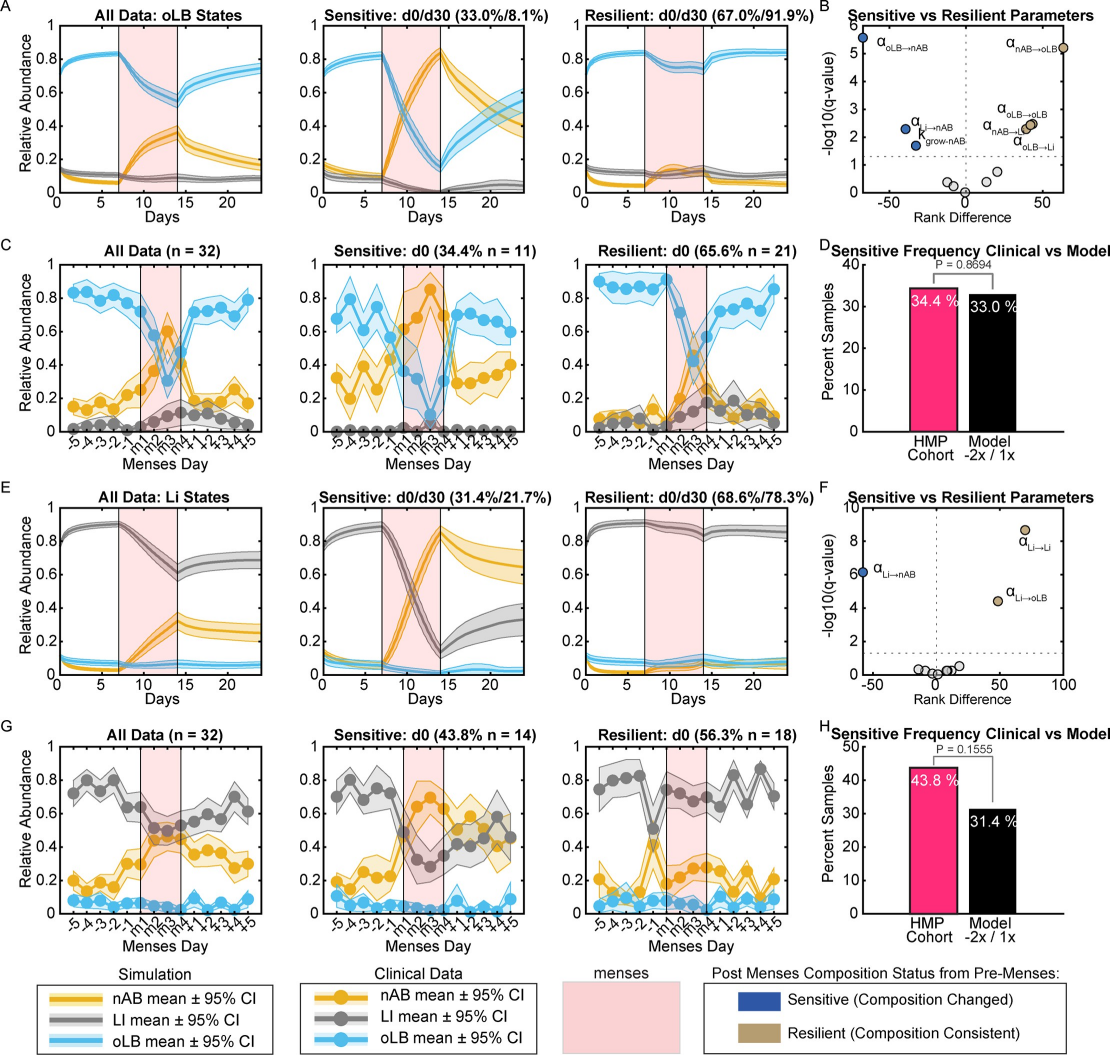
Menses-associated compositional fluctuations *in silico* and in clinical samples. The analysis was stratified dependent on the *Lactobacillus* spp. that was dominant pre-menses (A-D) are associated with oLB dominated equilibrium behavior (1SS oLB dominated and 2SS oLB dominated/nAB dominated) and (E-H) are associated with Li dominated equilibrium behavior (1SS Li dominated and 2SS Li dominated/nAB dominated subtypes). (A) Mean and ± 95% confidence interval of model predicted composition before, during (red), and after menses for nAB, Li, and oLB relative abundance. Data are plotted in aggregate (all data) and stratified by composition on the last day of menses. Samples that were nAB became dominant by the last day of menses were considered sensitive (middle) and those that remained *Lactobacillus* spp. dominant were considered resilient (right). (B) Mean and ± 95% confidence interval of nAB, Li, and oLB relative abundance for the HMP cohort data five days before menses, four representative time points during menses, and five days after menses. Data are plotted by aggregate and response types as described in panel A. (C) Volcano plot comparing parameter differences between the sensitive (blue) and resilient (gold) response types from the model simulation. (D) Comparison of clinical versus model predictions for the frequency of menses-sensitive samples. (E-H) Corresponding analysis for the Li dominated states (E) Model simulations in aggregate and stratified by response type (F) Clinical observations in aggregate and stratified by response type. (G) Volcano plot of parameters that differ between response types. (H) Comparison of clinical versus model predictions for the frequency of menses-sensitive samples. Statistical comparisons of frequency were made using χ2-tests. The magnitude of menses perturbation in this figure was -200% k_grow-oLB_/kgrow-Li and +100% α_oLB→nAB_/α_oLi→nAB_.

Since the bifurcation analyses assume sustained alterations in parameters, it does not necessarily demonstrate mechanisms for dynamic and dramatic changes that can occur due to temporary perturbations such as menses. Thus, a simulated seven-day menses was completed at four different magnitudes ranging from a 50% decrease in k_grow-Li_ and k_grow-oLB_ and 50% increase in α_Li→nAB_, α_oLB→nAB_ to a 200% decrease in k_grow-Li_ and k_grow-oLB_ and 100% increase in α_Li→nAB_, α_oLB→nAB_ (S4 and S5 Figs). Four magnitudes were evaluated because of uncertainty on how strongly menses impacts vaginal microbiota *in vivo*. Of the simulated magnitudes, the perturbation that was most similar to the HMP clinical cohort was the 200% decrease in k_grow-Li_ and k_grow-oLB_ and 100% increase in α_Li→nAB_, α_oLB→nAB_ which was further evaluated for patients that were oLB dominated or Li dominated pre-menses (Fig 4). For samples with equilibrium behaviors that are oLB dominant (1SS oLB dominated and 2SS oLB dominated/nAB dominated) the average sample exhibited a transient decrease in oLB abundance during menses (Fig 4A, left). Of the oLB dominated equilibrium behavior samples, 33.0% exhibited nAB dominance on the last day of menses (day 0, resilient group) and 8.1% of simulated samples exhibiting sustained nAB dominance at 1 month (day 30, Fig 4A, middle). Lastly, a subset of samples did not undergo a shift to nAB dominance as evaluated on the last day of menses (resilient group, Fig 4A, right). To identify microbial characteristics driving the differences in response to menses, multiple Wilcoxon Rank Sum tests were completed to compare parameters associated with sensitive versus resilient menses response groups (Fig 4B). Drivers of sensitivity were the growth rate of the nAB species (k_grow-nAB_) and the strength of oLB and Li inhibition of the nAB species (α_oLB→nAB_ α_Li→nAB_). In contrast, the interaction of the nAB species on oLB (α_nAB→oLB_) was associated with resilience and stability. This parameter is an understudied interaction in vaginal communities as most research focuses on the inhibitory properties of oLB on nAB species when assessing probiotics and vaginal ecology [18]. Menses data from the HMP cohort were categorized by equilibrium behavior subtype and visualized in a comparable manner to the simulations, demonstrating similar trends in composition fluctuations due to menses (Fig 4C). Evaluation of the frequency of communities that switched to nAB dominance after menses was not statistically different between the model predicted and clinically observed frequency (Fig 4D, 33.0% versus 34.4%, P = 0.8694). This analysis was repeated for the Li dominated equilibrium states (1SS Li dominated and 2SS Li dominated/ nAB dominated). Like the oLB states, on average Li abundance underwent transient composition shifts over time (Fig 4E, left). Of the simulated samples, 31.4% underwent a switch to nAB dominance as evaluated on the last day of menses (day 0, sensitive group, Fig 4E, middle) and 21.7% underwent sustained switches to nAB dominance (day 30). The remaining 68.6% of samples did not undergo a switch to nAB dominance at the last day of menses (resilient group, Fig 4E, right). Comparison of parameter value differences by volcano plot implicated the importance of Li on nAB (α_Li→nAB_) and Li on oLB (α_Li→oLB_) with the response groups, highlighting the need to better understand the relationship of Li with facilitating or inhibiting vaginal microbiota associated with health and BV states (Fig 4F). Clinical data for the Li states was visualized by the overall average abundances (Fig 4G, left), the subset of sensitive samples (middle) and the subset of resilient samples (right). Evaluation of the frequency of communities switched to nAB dominance after menses was comparable between the model predicted and clinically observed frequency (Fig 4H, 31.4% versus 43.8%, P = 0.1555). Overall, this assessment supports the use of this modeling framework to predict response types to menses and links microbial parameters that could be potential targets to promote stability of optimal composition.

### Variability in BV recurrence after antimicrobial therapy may be driven by differences in equilibrium subtypes

**Fig 5:**
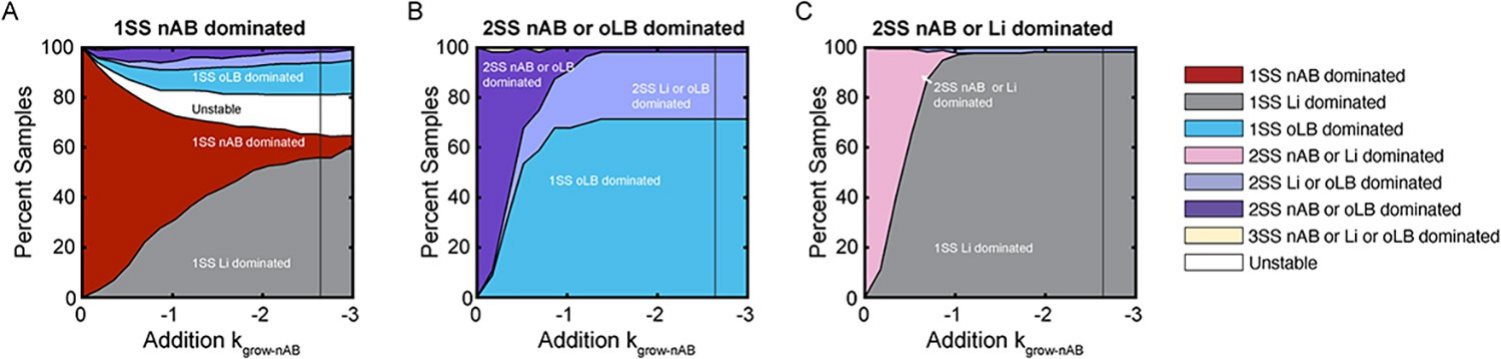
Bifurcation analysis to explore the impact of antibiotics on nAB dominated communities. One-dimensional bifurcation analysis altering k_grow-nAB_ to decrease to negative (death rates) to model antibiotic therapy. Colors indicate the equilibrium behavior subtype and the y-axis is the percent of samples at each given value of k_grow-nAB_ perturbation for (A) 1SS nAB dominated subtypes (B) 2SS nAB dominated/oLB dominated subtypes (C) 2SS nAB dominated/oLB dominated subtypes.

Antimicrobial therapies to treat bacterial vaginosis exhibit high rates of treatment failure, particularly recurrence [39]. To explore factors that can contribute to treatment failure, a bifurcation analysis and simulated course of antibiotics were completed for equilibrium behavior subtypes that can exhibit nAB dominance (1SS nAB dominated, 2SS nAB dominated/oLB dominated, 2SS nAB dominated/Li dominated). Like the menses analysis, the *in silico* HMP population was used to replicate expected frequencies of each equilibrium behavior subtype (S3 Fig). The bifurcation analysis, which explores sustained alterations mimicking antimicrobial therapy (inhibition of nAB growth, k_grow-nAB_) demonstrated that the 1SS nAB dominated communities required stronger inhibition to reach *Lactobacillus* spp. dominated equilibrium behavior subtypes (Fig 5A), whereas the 2SS nAB dominated/oLB dominated and 2SS nAB dominated/Li dominated communities switched to 1SS oLB dominated or 1SS Li dominated communities in greater than 50% of samples by the lowest decay rate reported in Mayer et al. 2015 (0.95 d^-1^; S4 Table and Fig 5B and 5C) [40].

**Fig 6:**
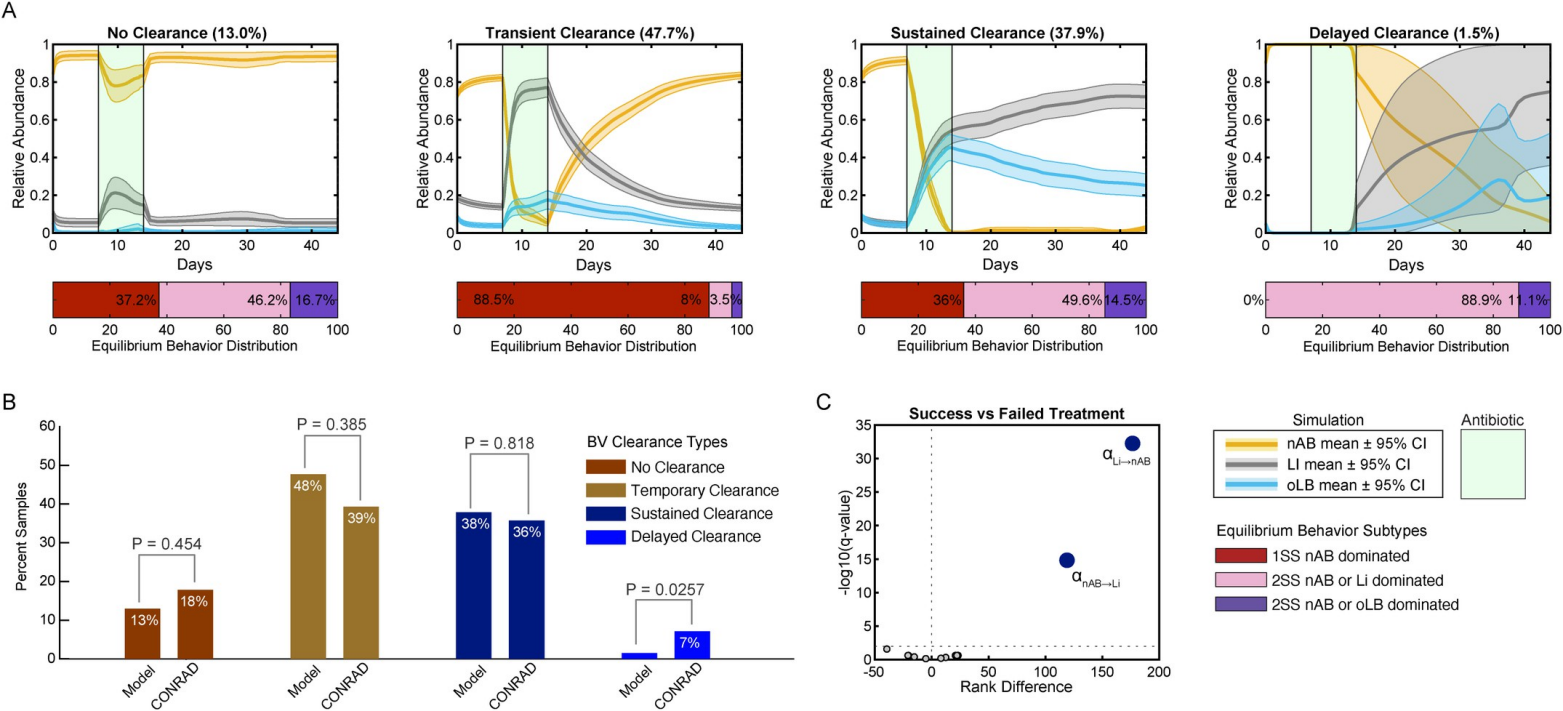
Predicted and clinically observed antibiotic response types. (A) Model predicted antibiotic response types. Each plot depicts the mean and ± 95% confidence interval for the relative abundance of nAB, Li, and oLB before antibiotic therapy, during antibiotic therapy (green), and the following month after therapy. The four plots represent trends observed in each response type (left to right): No response (no shift to oLB or Li dominance), temporary response (recurrence, initial oLB/Li dominance by last day of treatment, but returns to nAB dominance by 1 month), sustained response (cured, oLB/Li dominance at last day of treatment and 1 month later), and delayed response (nAB dominance at last day of treatment, oLB/Li dominance by 1 month). Below each plot is a breakdown of the percentage of equilibrium behaviors associated with each response type. (B) Comparison of model frequencies to the CONRAD BV cohort described in Gustin et al. 2022 (N = 28, χ2-tests) [40]. (C) Volcano plot exploring the parameter differences of model predicted treatment success group (sustained and delayed response) versus the treatment failure group (no response and recurrent response) samples using multiple Wilcoxon rank sum tests with FDR-adjusted p-values.

While the bifurcation analysis demonstrates a long-term result from sustained inhibition of nAB, traditional antibiotic therapies are a temporary, 5–7 day regimen. Additionally, efficacy of BV therapies is often assessed at different timepoints as individuals can exhibit no clearance of nAB during therapy (BV positive at therapy cessation and one month later), temporary clearance (BV negative at therapy cessation, BV positive at 1 month), delayed clearance (BV positive at therapy cessation, BV negative at 1 month), or sustained clearance (BV negative at therapy succession and 1 month post [41]). Thus, to understand the complexities that microbial characteristics can contribute to these variable response types, a 7-day course of antibiotic was simulated by changing the growth rate of nAB (k_grow-nAB_) to a decay rate. The decay rate was determined from clinical measurements of BV-associated bacteria abundance after a 5-day course of metronidazole gel (S4 Table, [40]). Simulations were assessed across three equilibrium behavior subtypes associated with BV, the mono-stable subtypes (1SS nAB dominated), and two types of multi-stable subtypes (2SS nAB dominated/Li dominated and 2SS nAB dominated/oLB dominated) at frequencies defined by the HMP cohort. Simulation results recapitulated all four clearance types reported for bacterial vaginosis, with 13.0% of samples exhibited no shift to oLB/Li dominance (no clearance), 47.7% of samples exhibiting a temporary shift to oLB/Li dominance (BV recurrence, temporary clearance), 37.9% exhibiting a sustained shift to oLB/Li dominance through 1 month post therapy (cured, sustained clearance), and 1.5% exhibiting no composition shift by the end of treatment, but oLB/Li dominance 1 month post (delayed clearance, Fig 6A). These results agreed well with a cohort of 28 women reported by Gustin et al. 2022 (BV CONRAD cohort), which reported 18% no clearance, 39% temporary clearance, 36% sustained clearance, and 7% delayed clearance (P = 0.454, P = 0.385, P = 0.818, P = 0.0257, respectively with model predictions; Fig 6B) [41]. Using the model to gain insight into microbial parameters that may drive differences in therapy success, model parameters in simulated samples that underwent successful treatment vs failed treatment by 1 month were evaluated with multiple Wilcoxon Rank Sum tests and visualized on a volcano plot (Fig 6C). The main drivers that differentiated response types were the pairwise interactions between nAB and Li (α_Li→nAB_, α_nAB→Li_), supporting the importance for better characterizing the relationship between Li and different species or strains of nAB. Notably, certain response types were associated with equilibrium behavior subtypes, particularly the transient BV clearance group suggesting recurrent BV may be driven by inherent microbial community stability to perturbations (Fig 6A).

### Combinatorial therapies and modified treatment duration demonstrate alternative strategies to treat BV

**Fig 7:**
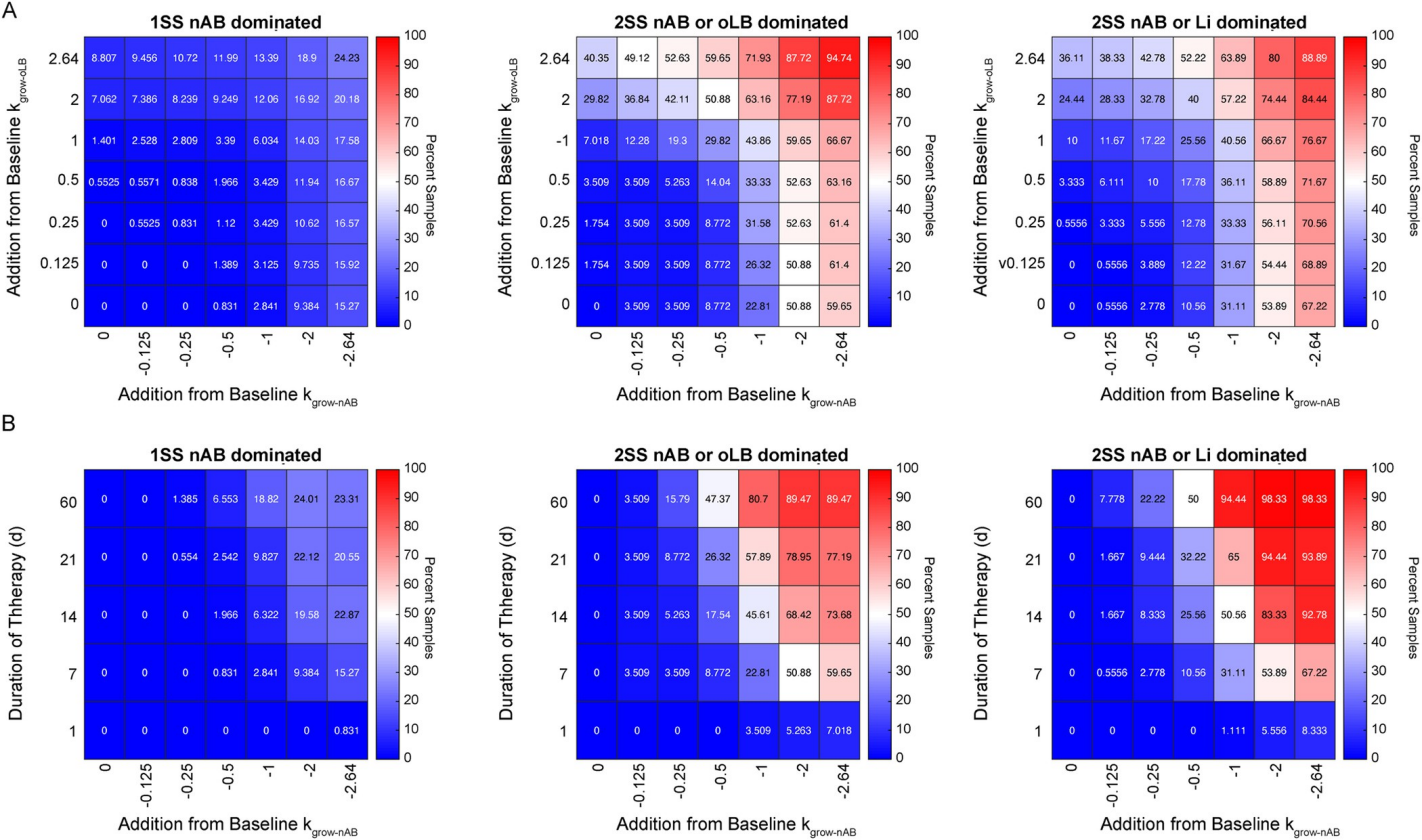
Simulated antibiotic regimens. (A) Combination antibiotic (k_grow-nAB_) and prebiotic (_kgrow-oLB_) across mono- and multi-stable subtypes. The percentage of parameter sets that underwent successful treatment 30 days after the regimen is displayed in the heatmap. (B) Alterations of antibiotic dose (k_grow-nAB_) and duration. The percentage of parameter sets that underwent successful treatment 30 days after the regimen is displayed in the heatmap.

To demonstrate how this framework could benefit the development of new BV therapies, the impact of combinations of an antibiotic and prebiotic and the relationship between dose and treatment duration on nAB clearance was evaluated. For the combination prebiotic (increase in k_grow-oLB_) and antibiotic (decrease in k_grow-nAB_), forty-nine combinations of doses were tested ranging from no change to a 2.64 d^-1^ increase or decrease (Fig 7A). As observed previously, the 1SS nAB dominated subtype had the lowest rates of successful treatment (decreased nAB relative abundance to less than 50% at day 30 post treatment; maximally 24.2%) compared to the multi-stable subtypes, which generally had higher success rates across all combinations of therapies (maximally 94.7% and 88.9% for the 2SS nAB dominated/oLB dominated and 2SS nAB dominated/Li dominated, respectively). Antibiotic impact on nAB growth rates was more important than prebiotic impact on oLB growth rates. Additionally, the maximum dose of only antibiotic had significantly higher success rates across the 1SS nAB dominated, 2SS nAB dominated/oLB dominated and 2SS nAB dominated/Li dominated subtypes compared to the maximum dose of only prebiotic (P = 0.0091, P = 1.175x10^-5^, P = 3.50x10^-9^). Similar trends were observed across the equilibrium subtypes for the dose-duration analyses where the 1SS nAB dominated had lower success rates (maximally 23.3%) compared to the 2SS nAB dominated/oLB dominated and the 2SS nAB dominated/Li dominated (maximally 89.5% and 98.3%, Fig 7B). Long-term antibiotic therapies (60 days) had similar efficacy in clearing BV at one month as the maximum dose combination of prebiotic and antibiotic across the three groups (1SS nAB dominated group: 23.3% vs 24.2%, P = 0.6389; 2SS nAB dominated/oLB dominated: 89.5% vs 94.7%, P = 0.0813; and 2SS nAB dominated/Li dominated: 98.3% vs 88.9%, P 0.0511). Overall, these results demonstrate that the model can be used to assess trade-offs between dose, duration, and the addition of new therapeutic strategies such as prebiotics.

## Discussion and conclusion

Here we show how linking ODE model equilibrium behavior with clinical VMB equilibrium behavior can give important insights into how variability in microbial growth characteristics and interactions between species may drive differences in clinical VMB stability and across women. This methodology overcomes challenges associated with the application of gLVMs to *in vivo* data, by using a top-down approach of matching parameter sets to observed equilibrium behaviors, rather than depending on direct parameter estimation from noisy, longitudinal data. This analysis adds another dimension to characterizing VMB CSTs, by characterizing temporal patterns in CSTs observed clinically. From this methodology, the model predicted similar frequencies of mono- and multi-stable subtypes as observed across two, ethnically diverse, clinical cohorts. These cohorts highlighted that vaginal microbiota communities exhibit three main CST-associated equilibrium behaviors: (1) Mono-stable optimal (1SS oLB dominated, 1SS Li dominated subtypes); (2) Mono-stable non-optimal (1SS nAB dominated subtype) and (3) Multi-stable (many combinations). The multi-stable subtypes, which comprised about a fifth of women, were observed across different combinations of CSTs, but were mainly associated with the 2SS Li dominated/nAB dominated subtype. The model additionally helped identify microbial factors that dictated differences between these equilibrium behavior subtypes, which could provide new targets to manage recurrent BV, such as nAB and Li interactions with oLB (*L*. *crispatus*, *L*. *jensenii*, *L*. *gasseri*). Overall, the results suggest modeling the VMB as an ecosystem with set equilibrium behaviors based on microbial growth characteristics and interactions can improve understanding of VMB dynamics and help identify target microbial characteristics to assess at a mechanistic level (such as by characterizing metabolic drivers behind these terms) to treat or prevent BV.

Menses simulations of healthy individuals pointed to the importance of *Lactobacillus* spp. inhibition nAB (α_oLB→nAB_, α_Li→nAB_) for samples that did not undergo alterations in composition to nAB dominance, suggesting supplementation of that interaction with treatments such as lactic acid could promote stability in healthy individuals [42]. Simulations of menses in non-optimal (CST -IV associated) individuals was not assessed. Though CST -IV associated communities also have associated compositional shifts, this study focused on healthy (oLB or Li dominated communities pre-menses) because the model does not differentiate between different species of within non-optimal communities and thus would not have the ability to capture these composition changes. The menses simulations replicated population level clinical observations in composition shifts from oLB dominated and Li dominated communities to nAB dominance (34.4% and 43.8% of clinical observations); however, this result was dependent on the strength of the simulated menses perturbation. There is limited knowledge on how and to what degree menses impacts microbial parameters (growth characteristics or interaction terms), and the degree of alteration studied here is likely an underestimation of how menses impacts vaginal microbiota as it is based on *in vitro* observations of select compounds associated with CST -IV and menses [38,43]. Additionally, other external factors are expected to repeatedly displace an individual’s vaginal microbiome from the community’s intrinsic equilibrium composition and, in combination with menses, would increase the likelihood of composition shifts. The combination of multiple external factors can be simulated in this framework and could help develop personalized models of VMB composition over time (S3 Table).

A major motivation of this study was to determine if it is possible for the VMB to exist in mono-stable or multi-stable equilibrium states and to provide insight into responses to antibiotic therapy. The model and clinical observations support that many women exist in a mono-stable nAB dominated state, which suggests that most women with BV will have difficulty successfully resolving the condition after a 5–7 day regimen of antibiotics, even if the BV-associated bacteria (nAB) are sensitive to the antibiotic. Model predictions on BV clearance types was comparable to a clinical cohort that followed patients over three visits (before treatment, 1 week post, 1 month post) and aligned with the high rates of recurrence reported in the literature (15–52%, 4 weeks or more after therapy [44]). In some individuals with a history of recurrent BV, alternative dosing regimens are recommended such as 750 mg metronidazole twice weekly for 3 months, but typically the benefits observed during treatment do not continue if the dosing is stopped [45]. Other regimens and new formulations have also been tested and have shown to prolong time to BV recurrence, but no one method appears to be completely effective [46–48]. Overall, the implication of the VMB as a primarily mono-stable system suggests that long-term treatments (antibiotics, probiotics, prebiotics, intravaginal gels with active agents that inhibit BV-associated bacterial growth) would be more effective for those with recurrent BV, as these systems require sustained alterations that impact microbial parameters such as growth rates or interaction terms to re-orient the system to a new state. Candidate parameters implicated by our model included inhibition of the growth of the nAB (k_grow-nAB_), and an understudied characteristic, the inhibition strength of nAB on oLB community members (α_nAB→oLB_). The former would relate to traditional inhibition of BV-associated bacteria growth rates with long-term antibiotic treatments or other therapies inhibiting BV-associated bacteria growth (such as lactic acid containing products and boric acid) and the latter points to potential alternative strategies to re-condition the cervicovaginal microenvironment by decreasing the ability of nAB to compete with oLB, which is likely mediated by metabolic phenotypes that are still poorly understood [35,36,49,50]. Additionally, the response of multi-stable subtypes to antibiotic therapy indicated the importance of Li, both in its interaction with the oLB and nAB, suggesting competition between Li and oLB as well as cooperation with nAB could promote treatment failure. This duality is interesting, as reports indicate the Li phenotype can vary dependent on environmental and community contexts and may be a fulcrum point that leads to dramatic shifts in community composition [51]. Li is also of interest as a target during BV treatment, with recent publications suggesting elimination of Li promotes stable transition to more optimal *Lactobacillus* spp. dominance [34,37].

Lastly, the model was used to explore alternative regimens for BV therapy, including combinatorial therapies and alterations to antibiotic dose and duration. These simulations highlight how the model may be applied to identify treatments that are most efficacious across a population with unknown stability status or to identify personalized regimens given a known equilibrium behavior subtype. An equilibrium behavior subtype could be determined from a patient’s history, which could be as simple as known recurrent episodes of BV (1SS nAB dominated subtype) to more nuanced identification of equilibrium behavior based on longitudinal monitoring of VMB composition. Monitoring of VMB composition is becoming more accessible with companies tailored to personalized VMB characterization like Juno Bio and Evvy. Future developments of the model can also begin to characterize efficacy of probiotic therapies and the how the timing or probiotic therapy relative to the antibiotic regimen can impact predicted treatment efficacy [52]. Notably, from the context of our results and ecological theory, it will be important to characterize not only how the probiotic interacts with target, BV-associate bacteria (nAB), but also how a woman’s existing vaginal microbiota interacts with the probiotic, as communities can commonly exclude newly introduced species (competitive exclusion) [53,54]. While the latter is likely intractable clinically as women can vary significantly in terms of composition, species, and strains, it could be simulated to identify key characteristics that make a probiotic most likely to succeed across a multitude of different VMBs.

While developing a personalized predictive model of VMB dynamics may be challenging, the generation of representative models that recapitulate clinically observed equilibrium subtypes could provide insights for strategies to mitigate BV. By defining appropriate physiological ranges that capture interpersonal and intrapersonal variability, these models could be used to help develop therapies that are either effective in specific subtypes (mono-stable vs multi-stable) or are effective more universally across heterogenous populations. Future studies are warranted to better define parameters that dictate the growth and interactions between microbial species in the vagina, as well as how molecular host-microbiome interactions may contribute to system dynamics (i.e., role of host-provided nutrients such as mucus). Cervicovaginal fluid composition, which is impacted by host-hormone and immune responses, likely dictates substrates required for microbial growth and ability to cross-feed or produce compounds that regulate the growth of other microbial species [35,55]. Additionally, interactions between BV-associated bacteria (nAB) or *L*. *iners* with vaginal epithelial cells could impact microbiome dynamics, as both produce cytolysins that lyse VECs increasing available nutrients, such as glycogen, which is preferentially metabolized by select vaginal species [36,50,56,57]. Models with increased resolution into these interactions through microbial metabolism of preferred carbon sources could help identify mechanisms of microbial shifts at the molecular level and help define new strategies to regulate the VMB. Lastly, methodical analysis of the impact of host behavior (sexual, hygienic, diet, drugs) could help assess the generalizability of the findings to real-world applications.

## Methods

### Model construction

A generalized Lotka-Volterra model (gLVM) with three equations was used as the ordinary differential equation-based model. gLVMs include the growth rate of each species, the self-interaction term (contributes to carrying capacity) and inter-species interaction terms. Growth rates were always assumed to be positive when the system is not under any perturbation like menses of antibiotic therapy, self-interaction terms are assumed to always be negative and the inter-species interaction terms can be either positive or negative. For the three species model (oLB, Li or nAB), there are seven possible non-zero steady states. These seven states were related to clinical data using a nearest centroid classifier of the predicted relative abundances. The centroids were determined from VALENCIA (S2 Table) [31]. All model simulations were completed in MATLAB 2020b and are published at: https://doi.org/10.5281/zenodo.7569363.


$$\frac{d[nAB]}{dt}=k_{grow-nAB}[nAB]+\alpha_{nAB\to nAB}[nAB][nAB]+\alpha_{Li\to nAB}[nAB][Li]+\alpha_{oLB\to nAB}[nAB][oLB]$$

$$\frac{d[Li]}{dt}=k_{grow-Li}[Li]+\alpha_{Li\to Li}[Li][Li]+\alpha_{nAB\to Li}[Li][nAB]+\alpha_{oLB\to Li}[Li][oLB]$$

$$\frac{d[oLB]}{dt}=k_{grow-oLB}[oLB]+\alpha_{oLB\to oLB}[oLB][oLB]+\alpha_{nAB\to oLB}[oLB][nAB]+\alpha_{Li\to oLB}[oLB][Li]$$

### Parameter selection

Parameter values were selected based on experimental and empirical observations (S1 Table). Since many of these parameters are unknown or expected to be variable, parameter value ranges were used throughout the manuscript. These parameter ranges were either based on calculations from digitized data as reported in Lee *et al*. (2020) or on assumptions of directionality (positive or negative interaction) from calculations or empirical observation [7]. As the relative magnitude of inter-species interaction term relative to self-interaction term provides a normalized metric of interaction strength, maximum and minimum inter-species interaction terms were matched with experimental observations. By selecting a minimal self-interaction term of -0.04 time^-1^density^-1^ and a maximal inter-species interaction term of ±0.12 time^-1^density^-1^, a maximal ratio of ±30x inter-/self-interactions was observed which was matched to maximal inter-/self-interactions strengths estimated from *in vitro* observations of various *Lactobacillus* strains co-cultured with *G*. *vaginalis* or *Prevotella bivia* providing a maximal absolute ratio of 40x (S1 Fig). *In vivo* estimated gLV parameters were on the same order of magnitude for the maximal inter/self-interaction ratio, which was 11x. *In vitro* and *in vivo* time scales are reported to be different, so *in vitro* parameter values are in terms of hours and *in vivo* parameters are in terms of days where relative differences between parameters are maintained [26].

### Base in silico population

Latin Hypercube Sampling (LHS) was used to generate parameter sets that have biological feasibility. Briefly, LHS is a stratified sampling method that evenly samples across defined parameter distributions. The parameter distributions used in this publication were defined from uniform distributions with minimum and maximum values reported in S1 Table. Growth rates and inter-species interaction terms we set to range 10-fold based on calculate values from *in vitro* studies and ranged from 0.1–1.00 time^-1^ and the inter-species interaction terms ranged from -0.004 to -0.04 density^-1^time^-1^ (See S1 Table). Interspecies interaction terms ranged from -0.12–0.12 density^-1^time^-1^, except in the case of oLB on nAB, as many reports suggest this interaction is only negative (-0.12–0 density^-1^time^-1^). Then, each of the parameter sets (N = 5,000) was analytically assessed for steady state stability using local stability analysis, which determines which of the seven non-zero states are stable (S1 Text).

### In silico clinical cohorts

*In silico* patient populations for the clinical cohorts were generated from resampled parameters sets. First, the base *in silico* population was used to define probability distribution functions for each equilibrium behavior. Using the probability distribution functions defined from the base parameter sets that were associated with each behavior, 5,000 new parameter sets were generated to create a reference population (N = 30,000). Parameter sets were then selected at random from the reference population in proportions each to the equilibrium behaviors observed in the HMP cohort (N = 1,000). Compositional profiles were also matched based on the 95^th^ percentile of relative abundance observed for nAB, Li and oLB.

### Bifurcation analyses

For the two-dimensional bifurcation analysis, a base parameter set with known steady-state behavior was selected and two groups of parameters (growth rates versus inter-species interaction terms) were varied from that starting point over the combination of 50x50 parameter combinations. For each of the 2,500 parameter combinations the stability of the steady states was evaluated using local stability analyses. This process was repeated for all LHS parameter sets that had the same equilibrium behavior. For example, for all LHS sets that were 1SS oLB dominated 2,500 parameter combinations were calculated for each and the most frequently observed equilibrium behavior at each point was plotted. For the one-dimensional bifurcation analysis used to understand the impact of antibiotics on the system, the growth rate of nAB was decreased incrementally from no change (base parameter value) to the base parameter value minus 3.0 d^-1^. The frequency of equilibrium behaviors across samples for each parameter alteration was reported.

### Perturbation analyses

Perturbation analyses were completed to simulate menses and antibiotic therapy using the *in silico* HMP cohort with certain equilibrium behaviors. For the menses analyses, the 1SS oLB dominated, 2SS oLB dominated/nAB dominated, 1SS Li dominated, 2SS Li dominated/nAB dominated were assessed with a perturbation that decreased the growth rates for Li and oLB (k_grow-Li_ and k_grow-oLB_) as well as the interaction terms for oLB/Li on nAB (α_oLB→nAB_ and α_Li→nAB_) over four set menses indices where “p” indicates the original parameter value (control/no change: k_grow-Li_ and k_grow-oLB_ = p + 0.0 x p, α_oLB→nAB_ and α_Li→nAB_ = p + 0.0 x p; light: k_grow-Li_ and k_grow-oLB_ p– 0.5 x p, α_oLB→nAB_ and α_Li→nAB_ = p + 0.5 x p; moderate: k_grow-Li_ and k_grow-oLB_ p– 1.0 x p, α_oLB→nAB_ and α_Li→nAB_ = p + 1.0 x p; and strong: k_grow-Li_ and k_grow-oLB_ p– 2.0 x p, α_oLB→nAB_ and α_Li→nAB_ = p + 1.0 x p). The average trajectory ±95% confidence interval of all the parameter sets exhibiting sensitivity or resilience to the menses perturbation were plotted and the number of simulated samples that had switched to a BV state (nAB composition greater than 50%) at day 0 and 30 after menses completed was reported. Menses sensitive individuals were defined as shifting to nAB dominance evaluated on the last day of the simulated menses. Parameters differentiated sensitive versus resilient groups were compared using multiple Wilcoxon Rank Sum tests with FDR-adjusted p-values. Frequencies of menses sensitive individuals were compared with the strongest menses index to the clinical data using χ2-tests.

For the antibiotic simulations, the simulated samples analyzed were in the 1SS nAB dominated, 2SS nAB dominated/Li dominated and the 2SS nAB dominated/oLB dominated subtypes, with the perturbation modeled off a decrease in nAB growth rate (k_grow-nAB_ minus 2.64 d^-1^) calculated from digitized data in reporting shifts in BV associated bacteria (nAB) following antibiotic treatment clinically (S4 Table) [40]. The average trajectory ±95% confidence interval of all the parameter sets exhibiting sensitivity or resilience to the antibiotic perturbation were plotted and the number of simulated samples that had switched to a *Lactobacillus* spp. dominated state (nAB composition less than 50%) at day 0 and 30 after the antibiotic regimen was completed was reported. The frequency of four BV clearance profiles was analyzed and compared to reported frequencies in the CONRAD BV cohort based on the day 0 and day 30 post-antibiotic composition. Parameters differentiated sensitive versus resilient groups were compared using multiple Wilcoxon Rank Sum tests with FDR-adjusted p-values.

### Clinical datasets

The University of Maryland Baltimore, Human Microbiome Project (UMB-HMP) cohort data was previously published (Ravel et al., 2013 [12]) and all data provided was de-identified to this study. The original study was an observational prospective study, where treatment information was recorded daily by the participants and during a clinical exam at week 5 and week 10 for 135 nonpregnant women of reproductive age. Self-identified ethnicities of the 101 patients that met inclusion criteria (greater than 10 samples) were Black/African descent (60%), White/Caucasian (34%), Hispanic/Latina (5.0%), multi-racial (1%). Within this study, metronidazole treatment was provided as standard of care, as recommended by the CDC (Metronidazole 500 mg orally twice a day for 7 days) [44,58]. The original study protocol was approved by the Institutional Review Board of the University of Alabama at Birmingham and the University of Maryland School of Medicine. Written informed consent was appropriately obtained from all participants, who also provided consent for storage and use in future research studies related to women’s health. Patients self-collected cervicovaginal swabs for 10 weeks. Vaginal microbiota data was generated by sequencing the V3-V4 regions of the 16S rRNA gene and is available in dbGAP under BioProject PRJNA208535.

The Gajer cohort was previously published and data were downloaded from the supplementary files [11]. Briefly, the study collected longitudinal samples, twice weekly for 16 weeks in healthy reproductive-age women (N = 32) and quantified bacterial diversity using pyrosequencing of V1-V2 regions of 16S rRNA genes. Ethnicities of the 32 women included individuals that self-identified as White (41%), Black (50%), Hispanic (6%), or other (3%).

The CONRAD BV study has previously been described [41,59]. Patients with Nugent score of 4 or higher were screened across three visits, pre-treatment (visit 1), 7–10 days post-treatment (visit 2), and 28–32 days post-treatment (visit 3). Self-reported ethnicities in the original study included Hispanic White (3%), Hispanic Black (3%), Non-Hispanic Black (79%), Non-Hispanic White (6%), and mixed race (9%). Response types were characterized by patterns observed across the three visits, namely whether individuals improved to a *Lactobacillus* spp. dominated CST. Of the 28 patients, 25% failed to clear BV (no clearance), 35.7% exhibited transient clearance, 7.1% exhibited clearance at the final visit (delayed clearance), and 32.1% exhibited sustained clearance. These frequencies were compared with model predicted BV clearance profiles using χ2-tests tests.

The UMB-HMP and Gajer cohort data were assessed for multi-stability by analyzing patients who had greater than 10 sampled time points (HMP, N = 101; Gajer, N = 32). Each time point was converted to a CST type using the nearest centroid classifier described above (required conversion of relative abundance data to oLB, Li and nAB dominated groups). Using the classified CSTs at each time point, a matrix of state transitions was generated, which describes the frequency fluctuations in CST states, with individuals who are stable staying in “within” state CSTs. Transition matrices were then used to identify mono-stable vs multi-stable individuals using a nearest centroid classifier, where centroids were based at 100% within state transitions (mono-stable) and 50%/50% of pairwise CSTs (bi-stable). For analysis of menses transitions, the HMP cohort was used to analyze oLB dominated and Li dominated equilibrium behaviors. The time series data corresponding to these two categories were analyzed by extracting composition data for the five days prior, at least four days during, and five days post menses, for all occurrences of menstrual bleeding longer than 3 days. Frequencies of composition shifts were compared frequencies with model predicted composition shifts to nAB dominance using χ2-tests tests.

## Supporting information

S1 Fig Analysis of Inter-species/Self-interaction Parameter Ranges. The maximum magnitude of parameter ranges was set based on relative estimation of inter-species interaction terms to intra-species interaction terms, reaching ±30x using the selected parameter ranges in S1 Table (top left histogram). These ranges are on the same order of magnitude as gLV parameters estimated from *in vivo* gut microbiome experiments (Stein et al., 2013[26]; top right histogram). Interaction terms calculated from Atassi et al. 2006 [18] *in vitro* co-cultures observed high estimated ratios of inter/self-interaction terms, of up to 46x.
(DOCX)

S2 Fig Determination of Clinical Equilibrium Behavior Subtypes. (A-B) Example time series CST classifications from two patients in the HMP cohort and their respective state transition frequencies. State transition matrices display the frequency of switches across a time step, where combinations across the diagonal indicate the current time step and the next time step were in the same compositional state. (A) Example of a 1SS oLB dominated equilibrium behavior. (B) Example of 2SS nAB or oLB dominated equilibrium behavior. (C-D) Classification of each patient to an equilibrium behavior based on the frequency at which the patient remained within a transition state over each time step for (C) the HMP cohort (N = 101) and (D) the Gajer et al. cohort (N = 32) [11].
(DOCX)

S3 Fig Generating matched *in silico* populations to clinical data. (A) Workflow to create base population parameter sets from empirical observations for parameter ranges. (B) Creation of a reference population for each equilibrium behavior subtype. For example, the parameter sets generated in the base population that had 1SS oLB dominated equilibrium behavior were used to create a new probability distribution for each parameter to sample with Latin Hypercube Sampling. For each equilibrium behavior, 5000 parameter sets were selected from the equilibrium behavior specific probability distribution to create a reference population for each equilibrium type shown in panel (C). Lastly, parameter sets were randomly sampled at frequencies defined by clinical observations to create an *in silico* cohort tailored to a specific clinical cohort shown in (D).
(DOCX)

S4 Fig Menses simulations at varying degrees of simulated strength for oLB dominated states. In (A-D) plots indicate the average of all simulated samples (left), the average for a subset of samples that undergo a composition shift (middle), and the average for a subset that does not undergo a composition shift (right) and a volcano plot representing parameters that significantly differed in the sensitive and resilient samples. (A) The impact of no parameter change on samples used in the menses analysis (control). (B) The impact of a -0.5x fold addition to k_grow-Li_ and k_grow-oLB_ with a +0.5x folder addition to α_Li→nAB_ and α_oLB→nAB_ (light perturbation). (C) The impact of a -1x fold addition to k_grow-Li_ and k_grow-oLB_ with a +1x folder addition to α_Li→nAB_ and α_oLB→nAB_ (moderate perturbation). (D) The impact of a -2x fold addition to k_grow-Li_ and k_grow-oLB_ with a +1x folder addition to α_Li→nAB_ and α_oLB→nAB_ (strong perturbation). (E) Clinical observations for all samples (left), sensitive samples (middle) and resilient samples (right). (F) Statistical comparison of clinically observed sensitive sample frequency with model predicted frequencies at varying degrees of menses strength described in panels B-D. Statistical comparisons were made using χ^2^-tests.
(DOCX)

S5 Fig Menses simulations at varying degrees of simulated strength for Li dominated states. In (A-D) plots indicate the average of all simulated samples (left), the average for a subset of samples that undergo a composition shift (middle), and the average for a subset that does not undergo a composition shift (right) and a volcano plot representing parameters that significantly differed in the sensitive and resilient samples. (A) The impact of no parameter change on samples used in the menses analysis (control). (B) The impact of a -0.5x fold addition to k_grow-Li_ and k_grow-oLB_ with a +0.5x folder addition to α_Li→nAB_ and α_oLB→nAB_ (light perturbation). (C) The impact of a -1x fold addition to k_grow-Li_ and k_grow-oLB_ with a +1x folder addition to α_Li→nAB_ and α_oLB→nAB_ (moderate perturbation). (D) The impact of a -2x fold addition to k_grow-Li_ and k_grow-oLB_ with a +1x folder addition to α_Li→nAB_ and α_oLB→nAB_ (strong perturbation). (E) Clinical observations for all samples (left), sensitive samples (middle) and resilient samples (right). (F) Statistical comparison of clinically observed sensitive sample frequency with model predicted frequencies at varying degrees of menses strength described in panels B-D. Statistical comparisons were made using χ^2^-tests.
(DOCX)

S1 Table Explanation of LHS parameter ranges. Note the determination of inter-species interaction terms was based on empirical observation and hypothesis on interaction term strength and directionality. More information is in S1 Text.
(DOCX)

S2 Table Model CST centroids. (DOCX)

S3 Table Examples of how external factors can be simulated in the modeling framework. BV therapy and menses are simulated within this manuscript.
(DOCX)

S4 Table Calculated Antibiotic Impact on BV-associated bacteria (nAB). (DOCX)

S1 Text Analytical Determination of Steady-States. (DOCX)
