# Supplementary material for: Evaluation of vaginal microbiome equilibrium states identifies microbial parameters linked to resilience after menses and antibiotic therapy
Source: PLoS Comput Biol. 2023 Aug 11;19(8):e1011295. doi: 10.1371/journal.pcbi.1011295 (PMC10446192; doi:10.1371/journal.pcbi.1011295)
Supplement: S1 Fig — The maximum magnitude of parameter ranges was set based on relative estimation of inter-species interaction terms to intra-species interaction terms, reaching ±30x using the selected parameter ranges in S1 Table (top left histogram). These ranges are on the same order of magnitude as gLV parameters estimated from in vivo gut microbiome experiments (Stein et al., 2013[26]; top right histogram). Interaction terms calculated from Atassi et al. 2006 [18] in vitro co-cultures observed high estimated ratios of inter/self-interaction terms, of up to 46x. (DOCX) [file pcbi.1011295.s001.docx]

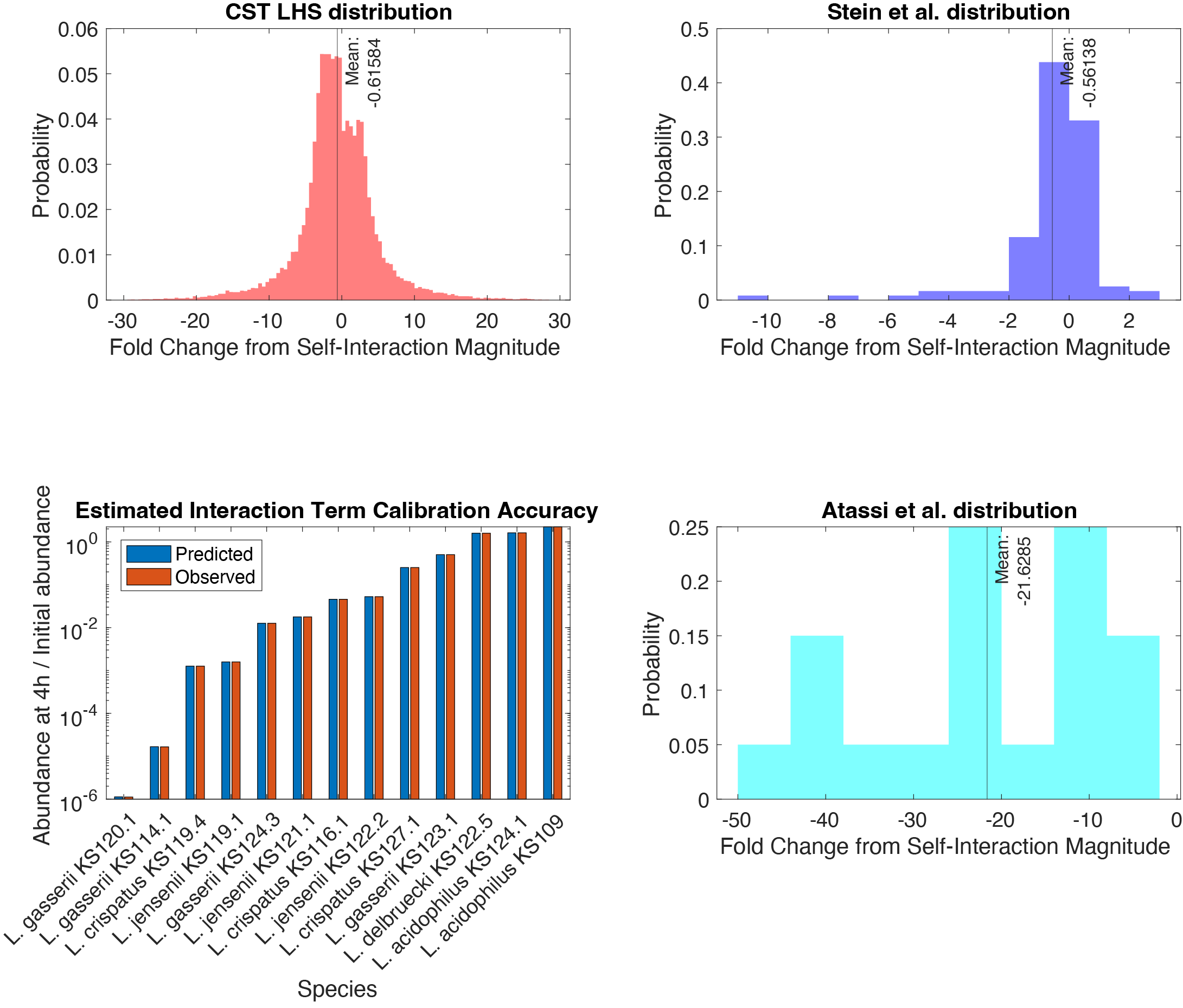


**S1 Fig. Analysis of Inter-species/Self-interaction Parameter Ranges.** The maximum magnitude of parameter ranges was set based on relative estimation of inter-species interaction terms to intra-species interaction terms, reaching ±30x using the selected parameter ranges in S1 Table (top left histogram). These ranges are on the same order of magnitude as gLV parameters estimated from *in vivo* gut microbiome experiments (Stein et al., 2013 [1]; top right histogram). Interaction terms calculated from Atassi et al. 2006 [2] *in vitro* co-cultures observed high estimated ratios of inter/self-interaction terms, of up to 46x.

**References**

1. Stein RR, Bucci V, Toussaint NC, Buffie CG, Rätsch G, Pamer EG, et al. Ecological modeling from time-series inference: insight into dynamics and stability of intestinal microbiota. PLoS Comput Biol. 2013;9: e1003388. doi:10.1371/journal.pcbi.1003388

2. Atassi F, Brassart D, Grob P, Graf F, Servin AL. Lactobacillus strains isolated from the vaginal microbiota of healthy women inhibit Prevotella bivia and Gardnerella vaginalis in coculture and cell culture. FEMS Immunol Med Microbiol. 2006;48: 424–432. doi:10.1111/j.1574-695X.2006.00162.x
