# Supplementary material for: Evaluation of vaginal microbiome equilibrium states identifies microbial parameters linked to resilience after menses and antibiotic therapy
Source: PLoS Comput Biol. 2023 Aug 11;19(8):e1011295. doi: 10.1371/journal.pcbi.1011295 (PMC10446192; doi:10.1371/journal.pcbi.1011295)
Supplement: S2 Fig — (A-B) Example time series CST classifications from two patients in the HMP cohort and their respective state transition frequencies. State transition matrices display the frequency of switches across a time step, where combinations across the diagonal indicate the current time step and the next time step were in the same compositional state. (A) Example of a 1SS oLB dominated equilibrium behavior. (B) Example of 2SS nAB or oLB dominated equilibrium behavior. (C-D) Classification of each patient to an equilibrium behavior based on the frequency at which the patient remained within a transition state over each time step for (C) the HMP cohort (N = 101) and (D) the Gajer et al. cohort (N = 32) [11]. (DOCX) [file pcbi.1011295.s002.docx]

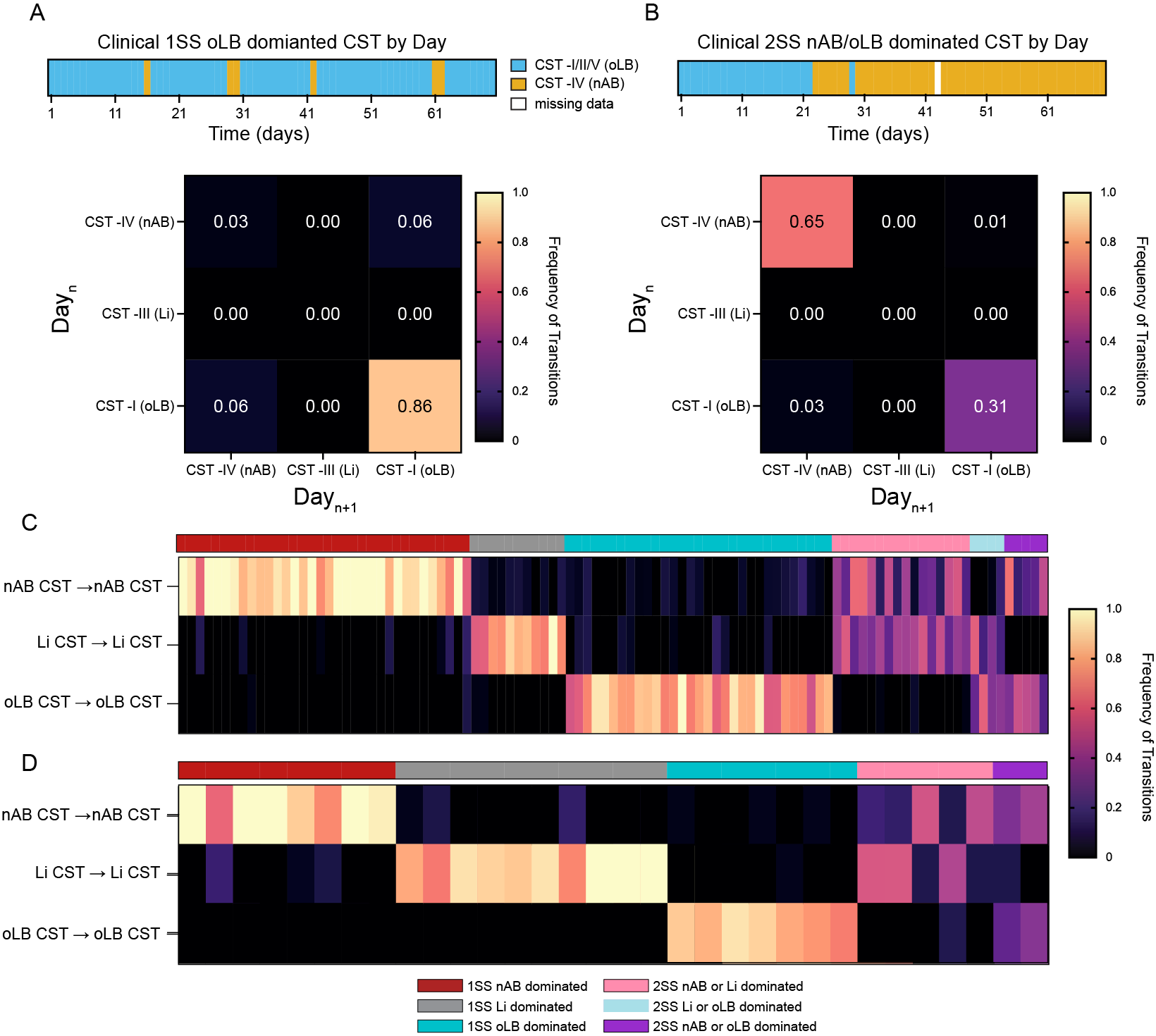


**S2 Fig. Determination of Clinical Equilibrium Behavior Subtypes.** (A-B) Example time series CST classifications from two patients in the HMP cohort and their respective state transition frequencies. State transition matrices display the frequency of switches across a time step, where combinations across the diagonal indicate the current time step and the next time step were in the same compositional state. (A) Example of a 1SS oLB dominated equilibrium behavior. (B) Example of 2SS nAB or oLB dominated equilibrium behavior. (C-D) Classification of each patient to an equilibrium behavior based on the frequency at which the patient remained within a transition state over each time step for (C) the HMP cohort (N = 101) and (D) the Gajer et al. cohort (N = 32) [1].

Reference

1. Gajer P, Brotman RM, Bai G, Sakamoto J, Schütte UME, Zhong X, et al. Temporal Dynamics of the Human Vaginal Microbiota. Sci Transl Med. 2012;4: 132ra52. doi:10.1126/scitranslmed.3003605
