# Supplementary material for: Evaluation of vaginal microbiome equilibrium states identifies microbial parameters linked to resilience after menses and antibiotic therapy
Source: PLoS Comput Biol. 2023 Aug 11;19(8):e1011295. doi: 10.1371/journal.pcbi.1011295 (PMC10446192; doi:10.1371/journal.pcbi.1011295)
Supplement: S3 Fig — (A) Workflow to create base population parameter sets from empirical observations for parameter ranges. (B) Creation of a reference population for each equilibrium behavior subtype. For example, the parameter sets generated in the base population that had 1SS oLB dominated equilibrium behavior were used to create a new probability distribution for each parameter to sample with Latin Hypercube Sampling. For each equilibrium behavior, 5000 parameter sets were selected from the equilibrium behavior specific probability distribution to create a reference population for each equilibrium type shown in panel (C). Lastly, parameter sets were randomly sampled at frequencies defined by clinical observations to create an in silico cohort tailored to a specific clinical cohort shown in (D). (DOCX) [file pcbi.1011295.s003.docx]

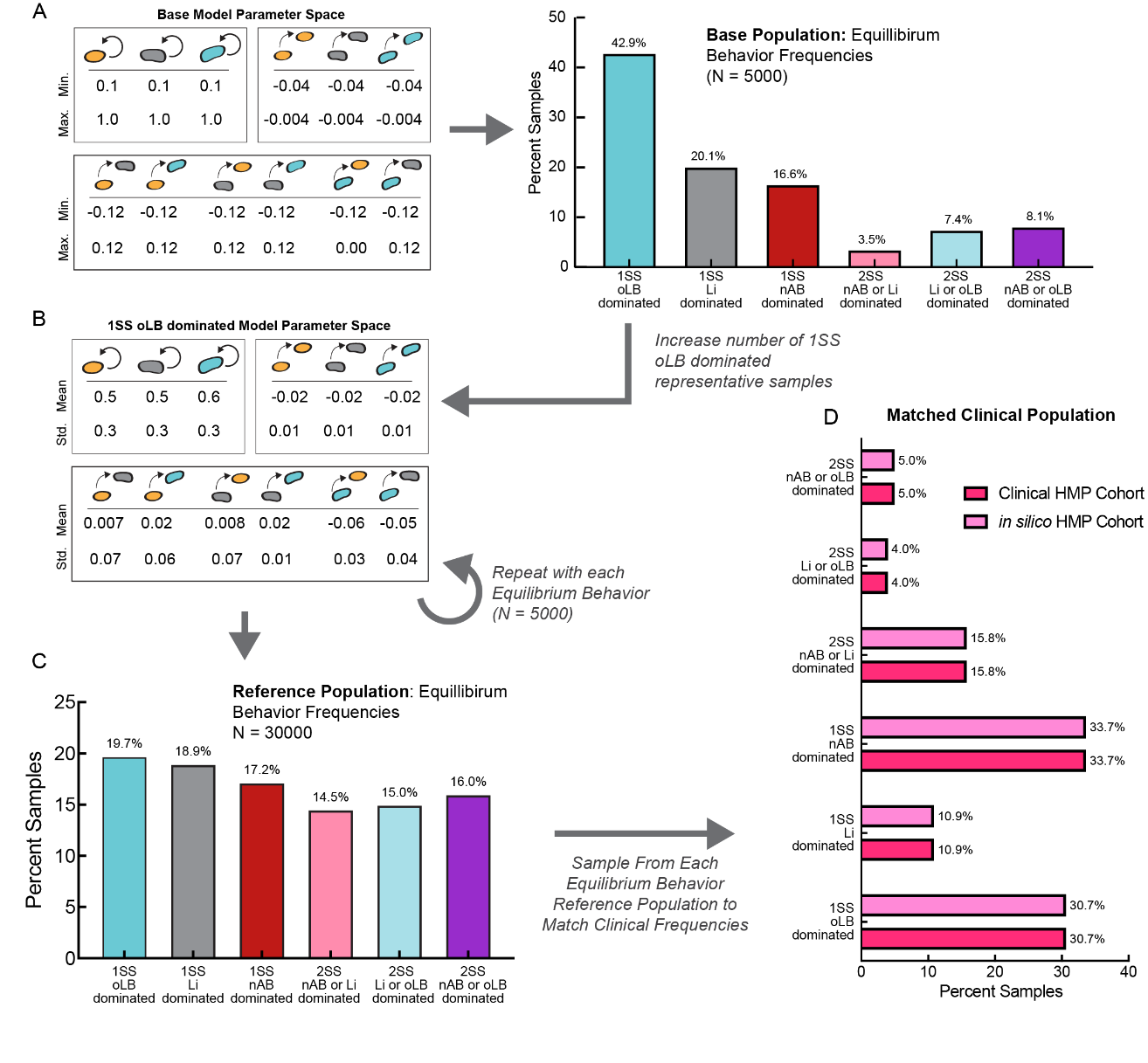


**S3 Fig. Generating matched *in silico* populations to clinical data.** (A) Workflow to create base population parameter sets from empirical observations for parameter ranges. (B) Creation of a reference population for each equilibrium behavior subtype. For example, the parameter sets generated in the base population that had 1SS oLB dominated equilibrium behavior were used to create a new probability distribution for each parameter to sample with Latin Hypercube Sampling. For each equilibrium behavior, 5000 parameter sets were selected from the equilibrium behavior specific probability distribution to create a reference population for each equilibrium type shown in panel (C). Lastly, parameter sets were randomly sampled at frequencies defined by clinical observations to create an *in silico* cohort tailored to a specific clinical cohort shown in (D).
