# Supplementary material for: Evaluation of vaginal microbiome equilibrium states identifies microbial parameters linked to resilience after menses and antibiotic therapy
Source: PLoS Comput Biol. 2023 Aug 11;19(8):e1011295. doi: 10.1371/journal.pcbi.1011295 (PMC10446192; doi:10.1371/journal.pcbi.1011295)
Supplement: S5 Fig — In (A-D) plots indicate the average of all simulated samples (left), the average for a subset of samples that undergo a composition shift (middle), and the average for a subset that does not undergo a composition shift (right) and a volcano plot representing parameters that significantly differed in the sensitive and resilient samples. (A) The impact of no parameter change on samples used in the menses analysis (control). (B) The impact of a -0.5x fold addition to kgrow-Li and kgrow-oLB with a +0.5x folder addition to αLi→nAB and αoLB→nAB (light perturbation). (C) The impact of a -1x fold addition to kgrow-Li and kgrow-oLB with a +1x folder addition to αLi→nAB and αoLB→nAB (moderate perturbation). (D) The impact of a -2x fold addition to kgrow-Li and kgrow-oLB with a +1x folder addition to αLi→nAB and αoLB→nAB (strong perturbation). (E) Clinical observations for all samples (left), sensitive samples (middle) and resilient samples (right). (F) Statistical comparison of clinically observed sensitive sample frequency with model predicted frequencies at varying degrees of menses strength described in panels B-D. Statistical comparisons were made using χ2-tests. (DOCX) [file pcbi.1011295.s005.docx]

**
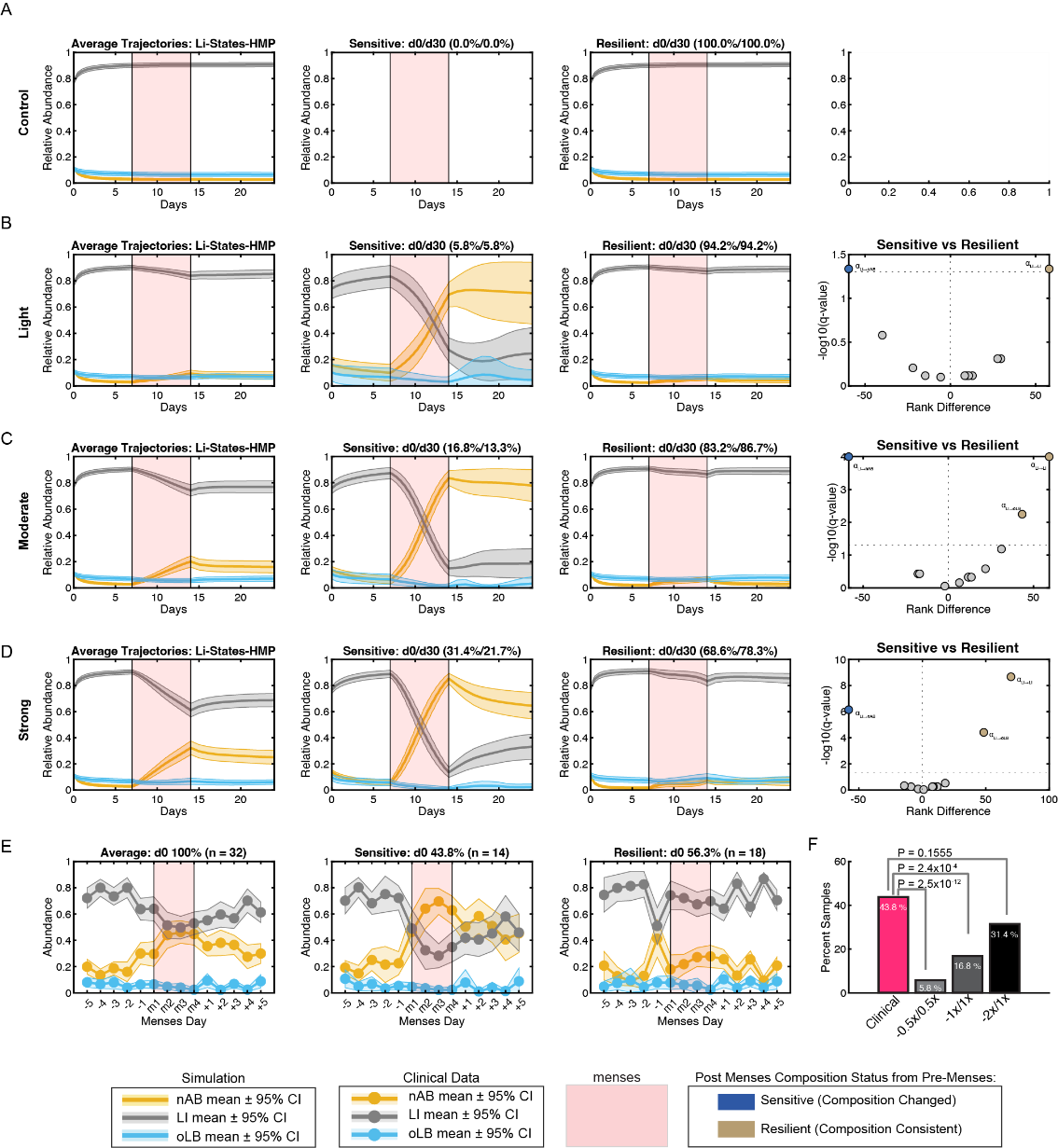
**

**S5 Fig. Menses simulations at varying degrees of simulated strength for Li dominated states.** In (A-D) plots indicate the average of all simulated samples (left), the average for a subset of samples that undergo a composition shift (middle), and the average for a subset that does not undergo a composition shift (right) and a volcano plot representing parameters that significantly differed in the sensitive and resilient samples. (A) The impact of no parameter change on samples used in the menses analysis (control). (B) The impact of a -0.5x fold addition to k_grow-Li_ and k_grow-oLB_ with a +0.5x folder addition to α_Li→nAB_ and α_oLB→nAB_ (light perturbation). (C) The impact of a -1x fold addition to k_grow-Li_ and k_grow-oLB_ with a +1x folder addition to α_Li→nAB_ and α_oLB→nAB_ (moderate perturbation). (D) The impact of a -2x fold addition to k_grow-Li_ and k_grow-oLB_ with a +1x folder addition to α_Li→nAB_ and α_oLB→nAB_ (strong perturbation). (E) Clinical observations for all samples (left), sensitive samples (middle) and resilient samples (right). (F) Statistical comparison of clinically observed sensitive sample frequency with model predicted frequencies at varying degrees of menses strength described in panels B-D. Statistical comparisons were made using χ^2^-tests.
