# Supplementary material for: Evaluation of vaginal microbiome equilibrium states identifies microbial parameters linked to resilience after menses and antibiotic therapy
Source: PLoS Comput Biol. 2023 Aug 11;19(8):e1011295. doi: 10.1371/journal.pcbi.1011295 (PMC10446192; doi:10.1371/journal.pcbi.1011295)
Supplement: S1 Table — Note the determination of inter-species interaction terms was based on empirical observation and hypothesis on interaction term strength and directionality. More information is in S1 Text. (DOCX) [file pcbi.1011295.s006.docx]

**S1 Table. Explanation of LHS parameter ranges.** Note the determination of inter-species interaction terms was based on empirical observation and hypothesis on interaction term strength and directionality. More information is in S1 Text.

| **Parameter** | **Value** | **Explanation** | **References** |
| --- | --- | --- | --- |
| k_grow-nAB_ (hr^-1^) | 0.1 to 1.00 | Growth rate calculated in previous publication from growth curves (Lee et al., 2020)[1] and assessed from digitized growth curves from literature such as Atassi et al., 2019[2] and Anukam and Reid (2008)[3]. | [1,3,4] |
| k_grow-Li_ (hr^-1^) | 0.1 to 1.00 | Growth rate calculated in previous publication from growth curves (Lee et al., 2020)[1] and from doubling times (Borgogna et al., 2021)[5]. | [1,5] |
| k_grow-oLB_ (hr^-1^) | 0.1 to 1.00 | Growth rate calculated in previous publication from growth curves (Lee et al., 2020) and from digitized data in Chetwin et al., 2019[6] and Juarez-Tomas (2003)[7] as well as Borgogna et al (2021)[5]. | [1,5,6,8] |
| α_nAB→ nAB_, α_Li → Li_, α_oLB → oLB_  (hr^-1^cell density^-1^) | -0.004 to  -0.04 | Assumed similar carrying capacities are possible across species and a 10-fold variability. This value and the growth rate value facilitate up to 100-fold variation in carry capacity and clinically relative abundance can upwards of range 1000-fold. | [9–11] |
| α_nAB → Li,_ α_nAB → oLB,_ α_Li → nAB,_ α_Li → oLB,_ α_oLB → Li_  (hr^-1^cell density^-1^) | -0.12 to  0.12 | Assumed directionally of these parameters to be positive or negative dependent on the literature. Magnitude of the values was determined from the largest ratio of interaction term to self-interact term observed experimentally (S1 Fig). The fold-ratio is on the same order of magnitude as clinically estimated gLV terms (Stein et al. 2013). | [12–14] |
| α_oLB → nAB_  (hr^-1^cell density^-1^) | -0.12 to  0.00 | Assume directionality based on experimental observations that oLB spp. commonly inhibit non-optimal spp. (nAB), such as in Atassi et al. (2006). | [2,13] |

**References**

1. Lee CY, Cheu RK, Lemke MM, Gustin AT, France MT, Hampel B, et al. Quantitative modeling predicts mechanistic links between pre-treatment microbiome composition and metronidazole efficacy in bacterial vaginosis. Nature Communications. 2020;11: 6147. doi:10.1038/s41467-020-19880-w

2. Atassi F, Brassart D, Grob P, Graf F, Servin AL. Lactobacillus strains isolated from the vaginal microbiota of healthy women inhibit Prevotella bivia and Gardnerella vaginalis in coculture and cell culture. FEMS Immunol Med Microbiol. 2006;48: 424–432. doi:10.1111/j.1574-695X.2006.00162.x

3. Anukam KC, Reid G. Effects of metronidazole on growth of Gardnerella vaginalis ATCC 14018, probiotic Lactobacillus rhamnosus GR-1 and vaginal isolate Lactobacillus plantarum KCA. Microbial Ecology in Health and Disease. 2008;20: 48–52. doi:10.1080/08910600701837964

4. Atassi F, Pho Viet Ahn DL, Lievin-Le Moal V. Diverse Expression of Antimicrobial Activities Against Bacterial Vaginosis and Urinary Tract Infection Pathogens by Cervicovaginal Microbiota Strains of Lactobacillus gasseri and Lactobacillus crispatus. Front Microbiol. 2019;10. doi:10.3389/fmicb.2019.02900

5. Borgogna J-LC, Shardell MD, Grace SG, Santori EK, Americus B, Li Z, et al. Biogenic Amines Increase the Odds of Bacterial Vaginosis and Affect the Growth and Lactic Acid Production by Vaginal Lactobacillus spp. Appl Environ Microbiol. 2021 [cited 10 Mar 2021]. doi:10.1128/AEM.03068-20

6. Chetwin E, Manhanzva MT, Abrahams AG, Froissart R, Gamieldien H, Jaspan H, et al. Antimicrobial and inflammatory properties of South African clinical Lactobacillus isolates and vaginal probiotics. Scientific Reports. 2019;9: 1917. doi:10.1038/s41598-018-38253-4

7. Juárez Tomás MS, Ocaña VS, Wiese B, Nader-Macías ME. Growth and lactic acid production by vaginal Lactobacillus acidophilus CRL 1259, and inhibition of uropathogenic Escherichia coli. J Med Microbiol. 2003;52: 1117–1124. doi:10.1099/jmm.0.05155-0

8. Tomás MSJ, Nader-Macías ME. Effect of a medium simulating vaginal fluid on the growth and expression of beneficial characteristics of potentially probiotic lactobacilli. Communicating Current Research and Educational Topics and Trends in Applied Microbiology,. 2007; 8.

9. Gajer P, Brotman RM, Bai G, Sakamoto J, Schütte UME, Zhong X, et al. Temporal Dynamics of the Human Vaginal Microbiota. Sci Transl Med. 2012;4: 132ra52. doi:10.1126/scitranslmed.3003605

10. Ravel J, Gajer P, Abdo Z, Schneider GM, Koenig SSK, McCulle SL, et al. Vaginal microbiome of reproductive-age women. PNAS. 2011;108: 4680–4687. doi:10.1073/pnas.1002611107

11. Ravel J, Brotman RM, Gajer P, Ma B, Nandy M, Fadrosh DW, et al. Daily temporal dynamics of vaginal microbiota before, during and after episodes of bacterial vaginosis. Microbiome. 2013;1: 1–6. doi:10.1186/2049-2618-1-29

12. Stein RR, Bucci V, Toussaint NC, Buffie CG, Rätsch G, Pamer EG, et al. Ecological modeling from time-series inference: insight into dynamics and stability of intestinal microbiota. PLoS Comput Biol. 2013;9: e1003388. doi:10.1371/journal.pcbi.1003388

13. Jackman CM, Deans KW, Forney LJ, Lin XN. Microdroplet co-cultivation and interaction characterization of human vaginal bacteria. Int Bio (Cam). 2019;11: 69–78. doi:10.1093/intbio/zyz006

14. Castro J, Henriques A, Machado A, Henriques M, Jefferson KK, Cerca N. Reciprocal Interference between Lactobacillus spp. and Gardnerella vaginalis on Initial Adherence to Epithelial Cells. Int J Med Sci. 2013;10: 1193–1198. doi:10.7150/ijms.6304
