# Supplementary material for: Evaluation of vaginal microbiome equilibrium states identifies microbial parameters linked to resilience after menses and antibiotic therapy
Source: PLoS Comput Biol. 2023 Aug 11;19(8):e1011295. doi: 10.1371/journal.pcbi.1011295 (PMC10446192; doi:10.1371/journal.pcbi.1011295)
Supplement: S2 Table — (DOCX) [file pcbi.1011295.s007.docx]

**S2 Table. Model CST centroids.**

|  | **Relative Abundance** | | |
| --- | --- | --- | --- |
|  | **nAB** | **Li** | **oLB** |
| **[nAB dominated] CST-IV** | 0.912 | 0.0592 | 0.0289 |
| **[Li dominated] CST-III** | 0.146 | 0.759 | 0.0946 |
| **[oLB dominated] CST -I/II/V** | 0.153 | 0.0952 | 0.752 |
