# Supplementary material for: Evaluation of vaginal microbiome equilibrium states identifies microbial parameters linked to resilience after menses and antibiotic therapy
Source: PLoS Comput Biol. 2023 Aug 11;19(8):e1011295. doi: 10.1371/journal.pcbi.1011295 (PMC10446192; doi:10.1371/journal.pcbi.1011295)
Supplement: S3 Table — BV therapy and menses are simulated within this manuscript. (DOCX) [file pcbi.1011295.s008.docx]

**S3 Table: Examples of how external factors can be simulated in the modeling framework.** BV therapy and menses are simulated within this manuscript.

| **External Factor** | **Methodology** | **Parameters Impacted** |
| --- | --- | --- |
| BV Therapy (Metronidazole or Clindamycin) | 1. Identified metronidazole impact on nAB is *in vivo*, from reported estimates on nAB population decay rates across multiple species with absolute abundance measurements [1] | 1. Population growth rate of nAB becomes negative (death rate), metronidazole is bactericidal |
| Menses | 1. Identified how menses impact vaginal microbial species (impact of increased biogenic amines associated with CST -IV and menses) | 1. Biogenic amines are associated with decreased growth rates *L. crispatus*, *L. gasseri*, *L. jensenii*, and *L. iners* as well as decreased D/L-lactic acid production [2] |
| Sexual Behavior / Partner Microbiome | 1. Model transfer of microbial species by “spiking in” microbial species into pre-existing *in silico* patient  2. Model the impact of increased pH associated with sexual activity (semen, lubricant, etc.) | 1. Alter abundance of model species at the frequency of sexual activity  2. Increased pH could alter growth rates (increase growth rate of nAB [3,4]) and decrease the impact of lactic acid/bacteriocins on nAB [5–7] |
| Hygienic Behavior | 1. Model loss or “wash out” of microbial species present in the pre-existing *in silico* patient  2. Model the impact of increased pH due to douching | 1. Alter abundance of model species at the frequency of douching  2. Increased pH could alter growth rates (increase growth rate of nAB [3,4]) and decrease the impact of lactic acid/bacteriocins on nAB [5–7] |
| Antifungal Therapy | 1. Would need to identify the impact of antifungals on microbial growth rates (reports are limited and some indicate that azithromycin, clotrimazole, or fluconazole have no substantial impact on *Lactobacillus* spp. [8,9]) | 1. Model change in microbial parameters (currently no *in vitro* data to support which parameters are impacted) |
| Contraceptives | 1. Identify impact of contraceptive on the vaginal microenvironment (e.g. glycogen and mucus levels)  2. Model competitive advantages gained by *Lactobacillus* spp. | Increased glycogen is associated with acidification of the vaginal that would promote stronger inhibition of nAB [10,11] |

**References**

1. Mayer BT, Srinivasan S, Fiedler TL, Marrazzo JM, Fredricks DN, Schiffer JT. Rapid and Profound Shifts in the Vaginal Microbiota Following Antibiotic Treatment for Bacterial Vaginosis. J Infect Dis. 2015;212: 793–802. doi:10.1093/infdis/jiv079

2. Borgogna J-LC, Shardell MD, Grace SG, Santori EK, Americus B, Li Z, et al. Biogenic Amines Increase the Odds of Bacterial Vaginosis and Affect the Growth and Lactic Acid Production by Vaginal Lactobacillus spp. Appl Environ Microbiol. 2021 [cited 10 Mar 2021]. doi:10.1128/AEM.03068-20

3. Shishpal P, Patel V, Singh D, Bhor VM. pH Stress Mediated Alteration in Protein Composition and Reduction in Cytotoxic Potential of Gardnerella vaginalis Membrane Vesicles. Front Microbiol. 2021;12: 723909. doi:10.3389/fmicb.2021.723909

4. Gottschick C, Szafranski SP, Kunze B, Sztajer H, Masur C, Abels C, et al. Screening of Compounds against Gardnerella vaginalis Biofilms. PLOS ONE. 2016;11: e0154086. doi:10.1371/journal.pone.0154086

5. Breshears LM, Edwards VL, Ravel J, Peterson ML. Lactobacillus crispatus inhibits growth of Gardnerella vaginalis and Neisseria gonorrhoeae on a porcine vaginal mucosa model. BMC Microbiol. 2015;15. doi:10.1186/s12866-015-0608-0

6. Atassi F, Brassart D, Grob P, Graf F, Servin AL. Lactobacillus strains isolated from the vaginal microbiota of healthy women inhibit Prevotella bivia and Gardnerella vaginalis in coculture and cell culture. FEMS Immunol Med Microbiol. 2006;48: 424–432. doi:10.1111/j.1574-695X.2006.00162.x

7. Atassi F, Pho Viet Ahn DL, Lievin-Le Moal V. Diverse Expression of Antimicrobial Activities Against Bacterial Vaginosis and Urinary Tract Infection Pathogens by Cervicovaginal Microbiota Strains of Lactobacillus gasseri and Lactobacillus crispatus. Front Microbiol. 2019;10. doi:10.3389/fmicb.2019.02900

8. Agnew KJ, Hillier SL. The effect of treatment regimens for vaginitis and cervicitis on vaginal colonization by lactobacilli. Sex Transm Dis. 1995;22: 269–273. doi:10.1097/00007435-199509000-00001

9. Ahrens P, Andersen LO, Lilje B, Johannesen TB, Dahl EG, Baig S, et al. Changes in the vaginal microbiota following antibiotic treatment for Mycoplasma genitalium, Chlamydia trachomatis and bacterial vaginosis. PLoS One. 2020;15: e0236036. doi:10.1371/journal.pone.0236036

10. Mirmonsef P, Hotton AL, Gilbert D, Burgad D, Landay A, Weber KM, et al. Free Glycogen in Vaginal Fluids Is Associated with Lactobacillus Colonization and Low Vaginal pH. PLoS One. 2014;9. doi:10.1371/journal.pone.0102467

11. Mirmonsef P, Hotton AL, Gilbert D, Gioia CJ, Maric D, Hope TJ, et al. Glycogen Levels in Undiluted Genital Fluid and Their Relationship to Vaginal pH, Estrogen, and Progesterone. PLOS ONE. 2016;11: e0153553. doi:10.1371/journal.pone.0153553
