# Supplementary material for: Evaluation of vaginal microbiome equilibrium states identifies microbial parameters linked to resilience after menses and antibiotic therapy
Source: PLoS Comput Biol. 2023 Aug 11;19(8):e1011295. doi: 10.1371/journal.pcbi.1011295 (PMC10446192; doi:10.1371/journal.pcbi.1011295)
Supplement: S4 Table — (DOCX) [file pcbi.1011295.s009.docx]

**S4 Table. Calculated Antibiotic Impact on BV-associated bacteria (nAB).**

| **Calculated Decay Rate (d^-1^)** | | | | | |
| --- | --- | --- | --- | --- | --- |
| ***G. vaginalis*** | **BVAB2** | **BVAB1** | ***Sneathia/Lepto*** | ***Megasphaera*** | ***A. vaginae*** |
| 3.82 | 4.61 | 3.50 | 5.04 | 4.08 | 5.44 |
| 3.12 | 2.15 | 2.26 | 4.18 | 4.08 | 4.81 |
| 2.45 | 2.15 | 2.26 | 3.19 | 3.88 | 4.81 |
| 2.12 | 2.15 | 2.08 | 2.87 | 2.76 | 3.80 |
| 1.84 | 1.88 |  | 2.41 | 2.03 | 3.48 |
| 1.72 | 1.69 |  | 2.13 | 2.03 | 3.23 |
| 1.40 | 1.66 |  | 2.07 | 2.03 | 2.38 |
| 1.33 |  |  | 1.82 | 1.74 | 1.88 |
| 1.33 |  |  | 1.73 |  | 1.88 |
| 0.95 |  |  | 1.44 |  | 1.54 |
