# Supplementary material for: Evaluation of vaginal microbiome equilibrium states identifies microbial parameters linked to resilience after menses and antibiotic therapy
Source: PLoS Comput Biol. 2023 Aug 11;19(8):e1011295. doi: 10.1371/journal.pcbi.1011295 (PMC10446192; doi:10.1371/journal.pcbi.1011295)
Supplement: S1 Text — (DOCX) [file pcbi.1011295.s010.docx]

**S1 Text.** **Analytical Determination of Steady-States**

*Steady-States:*

1. $\left( 0, 0, 0 \right)$
2. $\left( \frac{\alpha_{Li\to nAB}\mu_{Li} -\alpha_{Li\to Li}\mu_{nAB}}{\alpha_{nAB\to nAB}\alpha_{Li\to Li} - \alpha_{nAB\to Li}\alpha_{Li\to nAB}}, -\frac{\alpha_{nAB\to nAB}\mu_{Li}- \alpha_{nAB\to Li}\mu_{nAB}}{\alpha_{nAB\to nAB}\alpha_{Li\to Li} - \alpha_{nAB\to Li}\alpha_{Li\to nAB}}, 0 \right)$
3. $\left( \frac{\alpha_{oLB\to nAB}\mu_{oLB}- \alpha_{oLB\to oLB}\mu_{nAB}}{\alpha_{nAB\to nAB}\alpha_{oLB\to oLB} - \alpha_{nAB\to oLB}\alpha_{oLB\to nAB}},0, \frac{\alpha_{nAB\to nAB}\mu_{oLB} - \alpha_{nAB\to oLB}\mu_{nAB}}{\alpha_{nAB\to nAB}\alpha_{oLB\to oLB} -\alpha_{nAB\to oLB}\alpha_{oLB\to nAB}} \right)$
4. $\left( 0, \frac{\alpha_{oLB\to Li}\mu_{oLB} - \alpha_{oLB\to oLB}\mu_{Li}}{\alpha_{oLB\to oLB}\alpha_{Li\to Li} - \alpha_{oLB\to Li}\alpha_{Li\to oLB}}, -\frac{\alpha_{Li\to Li}\mu_{oLB} - \alpha_{Li\to oLB}\mu_{Li}}{\alpha_{oLB\to oLB}\alpha_{Li\to Li} - \alpha_{oLB\to Li}\alpha_{Li\to oLB}}, 0 \right)$
5. $\left( -\frac{\mu_{nAB}}{\alpha_{nAB\to nAB}}, 0, 0 \right)$
6. $\left( 0,-\frac{\mu_{Li}}{\alpha_{Li\to Li}}, 0 \right)$
7. $\left( 0, 0,-\frac{\mu_{oLB}}{\alpha_{oLB\to oLB}} \right)$
8. $\left( \begin{aligned} -\frac{\alpha_{Li\to nAB}\alpha_{oLB\to Li}\mu_{oLB} - \alpha_{nAB\to Li}\alpha_{oLB\to oLB}\mu_{oLi}+ \alpha_{Li\to Li}\alpha_{oLB\to oLB}\mu_{nAB} + \alpha_{Li\to oLB}\alpha_{oLB\to Li}\mu_{Li}- \alpha_{Li\to oLB}\alpha_{oLB\to Li}\mu_{nAB}}{\alpha_{nAB\to nAB}\alpha_{Li\to Li}\alpha_{oLB\to oLB}- \alpha_{nAB\to nAB}\alpha_{Li\to oLB}\alpha_{oLB\to Li}- \alpha_{nAB\to Li}\alpha_{Li\to nAB}\alpha_{oLB\to Li}+ \alpha_{nAB\to oLB}\alpha_{Li\to nAB}\alpha_{oLB\to Li}+ \alpha_{nAB\to oLB}\alpha_{Li\to Li}\alpha_{oLB\to nAB}}, \\ -\frac{\alpha_{Li\to nAB}\alpha_{oLB\to Li}\mu_{oLB} - \alpha_{Li\to nAB}\alpha_{oLB\to oLB}\mu_{Li}+ \alpha_{Li\to Li}\alpha_{oLB\to nAB}\mu_{oLB} + \alpha_{Li\to Li}\alpha_{oLB\to oLB}\mu_{nAB}- \alpha_{Li\to oLB}\alpha_{oLB\to Li}\mu_{nAB}}{\alpha_{nAB\to nAB}\alpha_{Li\to Li}\alpha_{oLB\to oLB}- \alpha_{nAB\to nAB}\alpha_{Li\to oLB}\alpha_{oLB\to Li}- \alpha_{nAB\to Li}\alpha_{Li\to nAB}\alpha_{oLB\to Li}+ \alpha_{nAB\to oLB}\alpha_{Li\to nAB}\alpha_{oLB\to Li}+ \alpha_{nAB\to oLB}\alpha_{Li\to Li}\alpha_{oLB\to nAB}} , \\ \frac{\alpha_{NO\to NO}\alpha_{oLB\to Li}\mu_{oLB} - \alpha_{NO\to NO}\alpha_{oLB\to oLB}\mu_{Li}+ \alpha_{NO\to Li}\alpha_{oLB\to oLB}\mu_{NO} + \alpha_{NO\to oLB}\alpha_{oLB\to NO}\mu_{Li}- \alpha_{NO\to oLB}\alpha_{oLB\to Li}\mu_{NO}}{\alpha_{nAB\to nAB}\alpha_{Li\to Li}\alpha_{oLB\to oLB}- \alpha_{nAB\to nAB}\alpha_{Li\to oLB}\alpha_{oLB\to Li}- \alpha_{nAB\to Li}\alpha_{Li\to nAB}\alpha_{oLB\to Li}+ \alpha_{nAB\to oLB}\alpha_{Li\to nAB}\alpha_{oLB\to Li}+ \alpha_{nAB\to oLB}\alpha_{Li\to Li}\alpha_{oLB\to nAB}} \end{aligned} \right)$

*Example Stability Conditions:*

The 100% nAB state, the eigen values of the Jacobian must be less than zero. Therefore, given the below eigen values the effect of the existing species (nAB) on the eliminated species (Li and oLB) must be negative.

$$\lambda_{1}= \mu_{Li}+ \alpha_{nAB\to Li}\left[ nAB \right]$$

$$\lambda_{2}= \mu_{oLB}+ \alpha_{nAB\to oLB}\left[ nAB \right]$$

$$\lambda_{3}= \mu_{NO}+ 2\alpha_{nAB\to nAB}[nAB]$$

The mixed nAB and Li state eigen values are more complicated and suggest the growth rate of the excluded species contributes to stability of this state, as well as at least one of the co-existing communities (nAB or Li) must inhibit the excluded species (oLB). The second eigen value also indicates the interactions between the co-existing species contribute to the stability of this state.

$$\lambda_{1}= \mu_{oLB}+ \alpha_{nAB\to oLB}\left[ nAB \right] + \alpha_{Li\to oLB}\left[ Li \right]$$

$$\lambda_{2/3}= \frac{\alpha_{nAB\to nAB}\left[ nAB \right]+\alpha_{Li\to Li}[Li]}{2} \pm\frac{\sqrt{\alpha_{nAB\to nAB}^{2}{[nAB]}^{2}-2\alpha_{nAB\to nAB}\alpha_{Li\to Li}\left[ nAB \right]\left[ Li \right]+ \alpha_{Li\to Li}^{2}\left[ Li \right]^{2}+4\alpha_{nAB\to Li}\alpha_{Li\to nABnAB}[nAB][Li]}}{2}$$
